# Burden of illness associated with eosinophilic granulomatosis with polyangiitis: a systematic literature review and meta-analysis

**DOI:** 10.1007/s10067-021-05783-8

**Published:** 2021-06-23

**Authors:** Rupert W. Jakes, Namhee Kwon, Beth Nordstrom, Rebecca Goulding, Kyle Fahrbach, Jialu Tarpey, Melissa K. Van Dyke

**Affiliations:** 1 Epidemiology, GSK, 980 Great West Road, London, TW8 9GS UK; 2 Respiratory Research & Development, GSK, London, UK; 3 Evidera, Boston, MA USA; 4 Epidemiology, GSK, Collegeville, PA USA

**Keywords:** Burden, Eosinophilic granulomatosis with polyangiitis, Incidence, Meta-analysis, Prevalence, Systematic review

## Abstract

Eosinophilic granulomatosis with polyangiitis (EGPA) is a rare disease associated with vascular inflammation and multisystem organ damage. The literature reporting EGPA incidence or prevalence is limited. We performed a systematic literature review and meta-analysis to describe the incidence, prevalence, and disease burden associated with EGPA. Real-world, observational, English-language studies in MEDLINE, MEDLINE In-Process, and Embase up to 6 June, 2019, were included. A single investigator screened all identified titles/abstracts and extracted data; an additional, independent investigator repeated the screening and validated the extracted data. A random-effects meta-analysis was conducted to generate pooled estimates for EGPA incidence and prevalence. Data from 100 eligible publications were extracted (32 with incidence/prevalence data, 65 with morbidity/healthcare resource data; 3 with both types of data). Significant evidence of between-study heterogeneity for reported incidence (p = 0.0013–0.0016) and prevalence (p = 0.0001–0.0006) estimates was observed. Global and European pooled estimates (95% confidence interval) of EGPA incidence were 1.22 (0.93, 1.60) and 1.07 (0.94, 1.35) cases per million person-years, respectively; global and European pooled estimates (95% confidence interval) for EGPA prevalence were 15.27 (11.89, 19.61) and 12.13 (6.98, 21.06) cases per million individuals, respectively. The proportions of patients experiencing relapses, or who had nasal polyps or severe asthma, varied considerably across studies. EGPA healthcare resource use was high, with inpatient admissions and emergency department visits reported for 17–42% and 25–42% of patients, respectively. Our results indicate that although global and European EGPA incidence and prevalence is low, the associated disease burden is substantial.

**Table:** 

			**Key points** • *We performed a systematic literature review and meta-analysis of real-world, observational studies describing the incidence, prevalence, and disease burden associated with eosinophilic granulomatosis with polyangiitis (EGPA).* • *Based on meta-analysis data from 35 eligible studies reporting incidence and prevalence, the incidence and prevalence of EGPA were low (globally 1.22 cases per million person-years and 15.27 cases per million individuals, respectively).* • *Among the 49 studies with morbidity and/or healthcare resource data, most reported a large proportion of patients with EGPA relapses and comorbidities of nasal polyps and severe asthma.* • *Healthcare resource use was also high among patients with EGPA in these studies, with inpatient admissions and emergency department visits reported for 17–42% and 25–42% of patients, respectively. Taken together, these data indicate the substantial disease burden associated with EGPA.*

**Supplementary Information:**

The online version contains supplementary material available at 10.1007/s10067-021-05783-8.

## Introduction

Eosinophilic granulomatosis with polyangiitis (EGPA), previously referred to as Churg-Strauss syndrome, is a systemic necrotising vasculitis associated with blood and tissue eosinophilia, extravascular granulomas, and asthma [1]. Other common characteristics of EGPA include sinusitis, pulmonary infiltrates, and neuropathy [2]. EGPA has a relapsing, remitting disease course, with an estimated 35% of patients relapsing less than 5 years after they achieve initial remission [3]. Available treatments for EGPA aim to minimise relapses and healthcare resource use (HCRU) for patients and typically consist of corticosteroids and/or immunosuppressive therapies. However, the long-term use of these drugs is associated with significant negative side effects [4, 5].

Although it is accepted that EGPA is a rare disease, there is limited literature reporting its incidence and prevalence. Several different types of nomenclature and diagnostic criteria, in addition to International Classification of Diseases (ICD) coding, can be used for EGPA identification and diagnosis, which make both diagnosing and estimating the incidence and prevalence of EGPA difficult. The Lanham diagnostic criteria, developed in 1984, require the presence of asthma, blood eosinophilia, and vasculitis involving two or more organs [6]. Subsequently, the American College of Rheumatology (ACR) developed its 1990 criteria [7], and the Chapel Hill Consensus Conference (CHCC) Nomenclature of Vasculitides was established in 1994 [8, 9]. The ACR guidelines are based on the presence of four out of six key criteria (asthma, eosinophilia, neuropathy, pulmonary infiltrates non-fixed, paranasal sinus abnormalities, and extravascular eosinophils) [7]. In contrast, the most recent (2012) CHCC nomenclature describe EGPA as eosinophil-rich and necrotising granulomatous inflammation, often involving the respiratory tract, as well as necrotising vasculitis predominantly affecting small to medium vessels and associated asthma and eosinophilia [9]. With a view to standardising EGPA diagnosis, the European Medicines Agency (EMA, formerly the European Medicines Evaluation Agency [EMEA]) more recently developed the EMEA 2007 algorithm: a stepwise algorithm that combines aspects of the Lanham criteria, ACR 1990 criteria, and CHCC 1994 nomenclature [10].

The objective of this systematic literature review was to describe the available published evidence on the incidence, prevalence, morbidity, and HCRU associated with EGPA. We also aimed to assess the heterogeneity in these outcomes that could be explained by country/region, population characteristics, and the diagnostic criteria/nomenclature used to identify patients with EGPA.

## Materials and methods

### Systematic literature review

#### Search strategy

The systematic literature review was designed to identify real-world, observational studies that reported data on EGPA incidence, prevalence and EGPA-associated morbidity, and HCRU. A literature search was carried out in MEDLINE, MEDLINE In-Process, and Embase for English-language studies published up to 6 June, 2019. The search strategies used a combination of controlled vocabulary and MeSH keywords, which were adjusted across databases. A detailed summary of the search criteria used can be found in Supplementary Tables 1 and 2. The Embase search also assessed conference abstracts from the two most recent key meetings of European Respiratory Society, American Thoracic Society, European League Against Rheumatology, and ACR, up to 6 June, 2019. An additional manual search of the bibliographies of published systematic literature reviews that were identified by this systematic literature review was undertaken for any supplementary materials that were not found via the database searches.

#### Selection criteria

Eligible publications (including abstracts) reported data for adults (≥ 18 years of age) with EGPA identified using one of the following validated diagnostic criteria/nomenclature or algorithms: Lanham 1984, ACR 1990, CHCC 1994/2012, EMEA 2007, ICD 9th revision (ICD-9) or 10th revision (ICD-10) codes for EGPA (ICD-9, 446.4; ICD-10, M30.1). Eligible publications also included data on the co-primary outcomes of interest, annual incidence and prevalence of EGPA (in European and non-European countries), and/or the exploratory outcomes of interest, morbidity (EGPA relapse, refractory EGPA, proportion of patients with nasal polyps [NP], and/or severe asthma), and HCRU (hospitalisation events, emergency department [ED] visits, specialist visits, and general practitioner visits). Only observational, real-world (prospective/retrospective cohort, cross-sectional, or case–control) studies were considered for inclusion; experimental studies were excluded. Only studies reported in the English language were included (Supplementary Table 3).

#### Study selection and data extraction

A single investigator used DistillerSR® software to screen all identified titles and abstracts; an additional, independent investigator performed a second round of screening and reviewed all excluded studies to confirm that the excluded publications and reasons for exclusion were correct. The full text articles of eligible records were reviewed by the same two reviewers. Any discrepancies identified during the abstract or full-text reviews were resolved by a third investigator. The study selection process was performed according to Preferred Reporting Items for Systematic Reviews and Meta-Analyses recommendations [11].

Once all suitable abstracts and full-text articles were identified, data from these publications were extracted for analysis by a single investigator. The data were then independently validated by an additional, independent investigator. In addition to the outcomes listed above, country, study design, date, patient age and sex, and sample size data were extracted to further assess the degree of heterogeneity. All secondary references and conference materials relating to a primary publication were reviewed to determine whether they contained any unique information that could be included. A third, independent investigator resolved any disagreements regarding data extraction and ensured consistency in the reporting of information across publications. The quality of each study was assessed using Joanna Briggs Institute Prevalence Critical Appraisal Tool [12]; the ten criteria used for this process are outlined in Supplementary Table 4.

#### Ethics

The analyses were based on publicly available summary-level data; ethical approval was therefore not required.

### Meta-analysis

A frequentist random-effects meta-analysis was conducted to generate pooled estimates for incidence and prevalence, globally (in all included studies) and in European countries, using the Metafor (2.1.0) package in R 3.5.3. This approach was deemed appropriate given that clinical, and methodological heterogeneity was likely to be observed across the included studies. Standard log and logit models were used for estimates of annual EGPA incidence and prevalence, respectively, and data presented as cases per million patient-years and cases per million patients, respectively. Where the data were available, period prevalence (estimated using the entire time period of a given study) and point prevalence (estimated at a single time point within a study) were also assessed. Interstudy heterogeneity was quantified using the Q-test of homogeneity and calculation of I^2^. In general, I^2^ values < 25% are considered minimal, ~ 50% are moderate, and > 75% are high.

## Results

### Study selection and characteristics of included studies

**Fig. 1:**
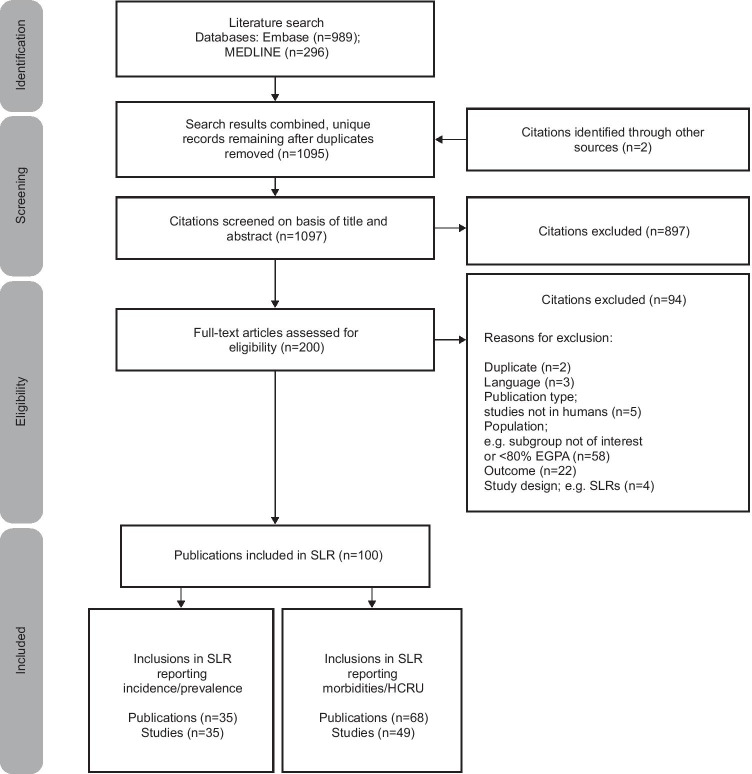
PRISMA flow diagram outlining the study selection process. Three publications (reporting the results of 3 studies) were included in both the incidence/prevalence reporting and the morbidities/HCRU reporting of the SLR. EGPA, eosinophilic granulomatosis with polyangiitis; HCRU, healthcare resource utilisation; PRISMA, Preferred Reporting Items for Systematic Reviews and Meta-Analyses; SLR, systematic literature review

In total, 1097 unique records were selected for full text screening based on their title and abstract. Following eligibility assessments, data from 100 publications were extracted. A total of 32 studies (reported in 32 publications) included annual incidence and/or prevalence data only; 46 studies (reported in 65 publications) included EGPA-associated morbidity and/or HCRU data only; 3 studies (reported in 3 publications) contained both annual incidence and/or prevalence data and EGPA-associated morbidity and/or HCRU data (Fig. 1). Most (n = 22) of the 35 included studies that reported incidence and prevalence data were retrospective observational studies; 9 were prospective cohort studies, and 4 were cross-sectional studies (Supplementary Table 4). The majority of incidence/prevalence studies were from Europe (n = 23), with 3 conducted in the USA; 4 conducted in multiple countries; and the remaining 5 studies conducted in Israel, Turkey, Australia, and Japan (Supplementary Table 4).

Of the 35 studies reporting incidence and prevalence data, 16 were included in the incidence analysis, providing 19 datapoints for different countries and time periods. Of the 19 excluded studies, 10 reported only prevalence data, and 9 studies reported incidence estimates from a data source used by one of the 16 included studies, but at earlier time points. A total of 13 studies were included in the prevalence analysis, 9 of which were included in the meta-analyses of period prevalence and four of which were included in the meta-analyses of point prevalence. Of the 22 excluded studies, 17 reported incidence data only, one was a prescription event monitoring study not considered representative of the general population, and four reported either a range in prevalence or prevalence estimates without details of the catchment population size.

We also observed significant evidence of between-study heterogeneity for reported incidence and prevalence estimates. The I^2^ values for incidence estimates were 66.05% (p = 0.0016) globally and 71.23% (p = 0.0013) in Europe. Global and European prevalence estimates were associated with I^2^ values of 90.56% (p = 0.0006) and 81.76% (p = 0.0001), respectively. We observed substantial heterogeneity in the criteria/nomenclature used to diagnose EGPA. In total, 13 out of 35 studies used single diagnostic criteria (ACR 1990: n = 7; CHCC 1994/2012: n = 5; ICD-10: n = 1). Ten studies used more than one criteria/nomenclature, most of which consisted of a combination of the ACR 1990 criteria and CHCC 1994 or 2012 nomenclature. Five studies reported using the EMEA 2007 algorithm. Finally, 7 studies did not report specifics regarding diagnostic criteria (Supplementary Table 4).

Among the 49 studies that presented data on EGPA-related morbidity and HCRU, 39 were retrospective studies. In addition, 4 were a hybrid of retrospective and prospective design, 2 were cross-sectional or cross-sectional/prospective studies, and 4 were prospective studies. The sample sizes of the included studies were generally small (consistent with the rare nature of the disease), with 41 studies having a patient sample size ≤ 50 (Supplementary Table 5). According to the risk-of-bias assessment, most (n = 36) studies reported appropriate recruitment methodologies (as per the Joanna Briggs Institute Prevalence Critical Appraisal Tool guidance [12]). However, only 14 studies described the patients and study settings in an appropriate level of detail, 18 studies reported appropriate statistical analysis methodology, and only 2 studies identified and accounted for confounding factors (Supplementary Table 6).

### Incidence of EGPA

**Fig. 2:**
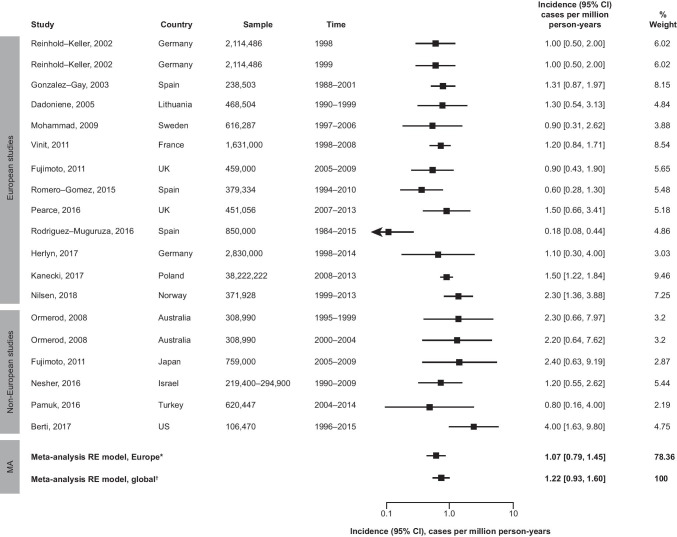
Estimated incidence of EGPA, globally and in Europe. * tau = 0.43, I^2^ = 71.23%, p = 0.0013; ^†^ tau = 0.43, I^2^ = 66.5%, p = 0.0016. The global meta-analysis RE model included all (European and non-European) studies; tau values describe the estimated standard deviation of underlying true effects across studies; I^2^ values represent the total proportion of variance in estimates that is due to heterogeneity. CI, confidence interval; EGPA, eosinophilic granulomatosis with polyangiitis; MA, meta-analysis; RE, random effects; UK, United Kingdom; US, United States

In Europe, EGPA incidence was lowest in Barcelona, Spain (0.18 cases per million person-years), and highest in Norway (2.50 cases per million person-years) (Fig. 2). Among other (non-European) studies, incidence was lowest in Turkey (0.80 cases per million person-years) and highest in the USA (4.00 cases per million person-years). There were no strong trends in incidence by sex; the annual incidence reported for male populations ranged from 0.6 cases per million person-years to 7.0 cases per million person-years, whilst the incidence in female subgroups ranged from 0.9 to 3.1 cases per million person-years. No strong trends in incidence over time, by country/region, or by diagnostic criteria were observed.

The global pooled estimate of EGPA incidence was 1.22 (95% confidence interval [CI]: 0.93, 1.60) cases per million person-years (Fig. 2), similar to the pooled estimate for Europe (1.07 cases per million person-years; 95% CI: 0.94, 1.35; Fig. 2). Incidence of EGPA in non-European countries ranged from 0.18 to 4.00 cases per million person-years.

### Prevalence of EGPA

**Fig. 3:**
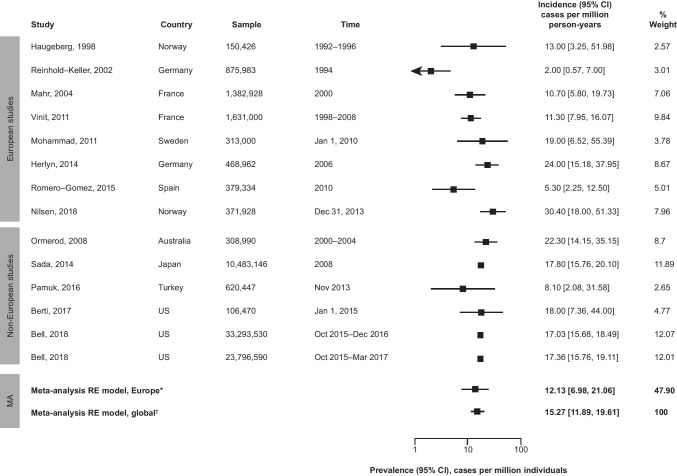
Estimated prevalence of EGPA, globally and in Europe. * tau = 0.68, I^2^ = 81.76%, p = 0.0001; ^†^ tau = 0.36, I^2^ = 90.56%, p = 0.0006. The global meta-analysis RE model included all (European and non-European) studies; tau values describe the estimated standard deviation of underlying true effects across studies; I^2^ values represent the total proportion of variance in estimates that is due to heterogeneity; the two prevalence estimates from Bell 2018 represent data from the same study collected separately from two different databases. CI, confidence interval; EGPA, eosinophilic granulomatosis with polyangiitis; MA, meta-analysis; RE, random effects

The global prevalence of EGPA ranged from 2.0 cases per million individuals in Germany to 30.4 cases per million individuals in Norway (Fig. 3). There were no strong trends in prevalence by sex; estimates ranged from 1.6 to 14 cases per million individuals for men and 6 to 14 cases per million individuals for women. No strong trends in prevalence over time, by country/region, or by diagnostic criteria were observed. The pooled estimate for EGPA prevalence (95% CI) was 15.27 (11.89, 19.61) cases per million individuals globally and 12.13 (6.98, 21.06) cases per million individuals in Europe (Fig. 3).

Period prevalence estimates (based on the entire time period of a given study) were reported in 9 out of the 13 studies with prevalence data. Point prevalence estimates (based on a single time point within a study) were reported in 4 studies; only 2 of these reported European data. Period prevalence estimates (95% CI) were 14.09 (10.47, 18.97) cases per million individuals globally and 9.54 (5.12, 17.78) cases per million individuals in Europe. Global and European point prevalence estimates (95% CI) were 21.18 (12.89, 34.79) and 27.76 (17.34, 44.44) cases per million individuals, respectively.

### EGPA-associated morbidity

A total of 45 studies reported morbidity data. From these, the percentage of patients with relapsed disease (typically defined as the new onset of clinical signs and symptoms attributable to active vasculitis) ranged from 6.1% (median 20 months of follow-up) to 81.1% (median 6 years of follow-up), with 18 of the 45 studies reporting relapse in ≥ 40% of patients. Among the 3 studies reporting the proportion of patients with refractory EGPA, estimates ranged from 2.3 to 20%.

Among the 20 studies reporting the occurrence of NP, 23–100% of patients with EGPA had NP, with 13 studies reporting NP in 40–60% of patients. The proportion of patients with severe asthma was reported in 18 studies, 5 of which defined asthma using GINA guidelines. According to these studies, 10–100% of patients with EGPA had severe asthma, with 11 studies reporting severe asthma in > 65% of patients.

### EGPA-associated HCRU

Three large claims database studies and three medical records studies reported EGPA-related HCRU. Among these, one study including data from two claims databases (reporting from 1 October 2015 to 31 December 2016 and 1 October 2015 to 31 March 2017) found that 25.1–33% of patients had an ED visit and 16.9–20.1% of patients had an inpatient admission. This same database study reported that 95% of patients had physician visits, 68% had hospital-based outpatient visits, and 99% had outpatient pharmacy prescriptions from 1 October 2015 to 31 March 2017. Another database study found that at 12 months post-index, 73% of patients had ambulatory visits, 42% had ED visits, and 29% had inpatient stays. Only 2 studies reported hospitalisations; one found that 42% of patients had an unscheduled hospital admission (from July 2010 to March 2013), and 1 study reported a median (range) of 1 (0–6) hospitalisation and 1 (0–12) ED visit per patient/year.

## Discussion

This is the first large-scale systematic literature review to investigate the incidence, prevalence, and disease burden of EGPA from the available literature. Based on meta-analysis data from 35 studies, global incidence and prevalence of EGPA were low (1.22 cases per million person-years and 15.27 cases per million individuals, respectively; 1.07 cases per million person-years and 12.13 cases per million individuals, respectively, in European countries). Although the specific incidence and prevalence values for EGPA differed between countries, incidence estimates were below 4 cases per million person-years and prevalence estimates below 31 cases per million individuals in all studies.

A key goal of treatment is to minimise relapses and HCRU for patients with EGPA. The proportions of patients with EGPA-associated morbidities varied considerably among studies. Nonetheless, 18 of the 45 studies with morbidity data reported that ≥ 40% of patients experienced EGPA relapses, 13 of the 20 studies reporting NP rates reported NP in between 40% and 60% of patients, and 11 of 18 studies reporting severe asthma rates found that > 65% of patients had severe asthma. Based on the six studies that presented data on HCRU, HCRU was high among patients with EGPA, with annual inpatient admissions and ED visits reported for 17–42% and 25–42% of patients with EGPA, respectively. These findings suggest that patients with EGPA experience a substantial disease burden in the form of high risk of relapse, associated morbidities, and frequent medical visits. They also suggest that during the time period included in this systematic literature review, there was a clear need for new treatment options to improve EGPA disease management. More recently, the humanised monoclonal antibody mepolizumab has been shown to increase accrued time in remission, increase the proportion of patients who achieve remission, increase time to first relapse, and reduce oral corticosteroid dose compared with placebo [2].

The synthesis of systematic literature review results via a meta-analysis requires sufficiently similar study and patient characteristics between the included studies. Among the included publications, there was evidence of statistically significant between-study heterogeneity for the incidence and prevalence estimates. We evaluated whether such heterogeneity could be explained by geographic location, diagnostic criteria, or patient age and sex. However, due to substantial study-level differences in sample size, study periods, and data sources, there was no strong evidence that incidence or prevalence of EGPA was associated with any of these factors. It was therefore not feasible to reliably evaluate potential trends in incidence or prevalence. A large real-world database study published after the time period of this systematic literature review has shown that in the USA, EGPA is more common in females than in males and more frequently reported in individuals over 50 years of age [13].

This study had several limitations. First, the included studies were observational, and therefore prone to bias and confounding. In addition, the protocol for this systematic literature review was not registered with the International Prospective Register of Systematic Reviews (PROSPERO); the prospective registration of protocols can assist in identifying any reporting biases. Systematic literature reviews are also inherently limited by the use of published data (and in this case the use of English-language publications only). We observed study-level heterogeneity in terms of population data sources, sample sizes, and study periods (both length of study and year of study), which impacted the overall reliability and generalisability of reported estimates. Moreover, historical data may not reflect current trends in EGPA incidence and prevalence, particularly since the nomenclature/criteria for identifying patients with EGPA has changed considerably over time. Indeed, we observed significant heterogeneity in the methods used to identify and diagnose EGPA; some studies used a combination of nomenclature/criteria, whilst others used a single item or did not describe the methods used. Heterogeneity was also observed between studies reporting morbidity and HCRU data: Some studies directly reported the proportion of patients with EGPA and refractory disease, NP, or asthma, whilst others assessed response to treatment for subgroups of patients stratified by the presence of these morbidities. Finally, the global pooled estimates of EGPA prevalence were strongly influenced by the results of one study (Bell, 2018) [14] that accounted for approximately 24% of the included data and reported an EGPA prevalence of 17.03–17.36 cases per million individuals from two US claims databases.

Overall, the data identified in this study suggest that EGPA has a low incidence and prevalence, globally and in Europe, consistent with the rare nature of the disease. Despite its rarity and with the current treatment options available, there appears to be a substantial morbidity and healthcare burden associated with EGPA, as indicated by the high risk of relapse, occurrence of NP and severe asthma, and high levels of HCRU. Our results also highlight a substantial variation in EGPA diagnostic criteria/nomenclature and methodologies used among the available published literature concerning the incidence, prevalence, and disease burden associated with EGPA.

## Supplementary Information

Below is the link to the electronic supplementary material.

Supplementary file1 (DOCX 254 KB)

## Data Availability

The analysable dataset is available from the authors upon request.

## Code availability

Exemplar code for a sample analysis can be provided upon request to the authors.
