# Supplementary material for: Burden of illness associated with eosinophilic granulomatosis with polyangiitis: a systematic literature review and meta-analysis
Source: Clin Rheumatol. 2021 Jun 23;40(12):4829–36. doi: 10.1007/s10067-021-05783-8 (PMC8599408; doi:10.1007/s10067-021-05783-8)
Supplement: Supplementary file 1 — Supplementary file1 (DOCX 254 KB) [file 10067_2021_5783_MOESM1_ESM.docx]

# Supplementary materials

**Burden of illness associated with eosinophilic granulomatosis with polyangiitis: a systematic literature review and meta-analysis**

Rupert W Jakes,^1^ Namhee Kwon,^2^ Beth Nordstrom,^3^ Rebecca Goulding,^3^ Kyle Fahrbach,^3^ Jialu Tarpey,^3^ Melissa K Van Dyke^4^

*^1^Epidemiology, GSK, London, UK; ^2^Respiratory Research & Development, GSK, London, UK; ^3^Evidera, Boston, USA; ^4^Epidemiology, GSK, Collegeville, USA*

**Corresponding author:**

Name: Rupert W Jakes

Address: Epidemiology, GSK, 980 Great West Road, London TW8 9GS

Tel: +44 20 8047 5000

Email: [rupert.2.jakes@gsk.com](mailto:rupert.2.jakes@gsk.com)

**Contents**

[Supplementary materials 1](#_Toc70591002)

[Table S1: Summary of MEDLINE and MEDLINE In-Process search terms (via PubMed) 2](#_Toc70591003)

[Table S2: Summary of EMBASE search terms 3](#_Toc70591004)

[Table S3: PICOS eligibility criteria 5](#_Toc70591005)

[Table S4: Summary of included incidence and prevalence studies 7](#_Toc70591006)

[Table S5: Summary of included morbidity and HCRU studies 13](#_Toc70591007)

[Table S6: Risk of bias quality assessment for incidence and prevalence studies 23](#_Toc70591008)

[List of publications included in the systematic literature review 26](#_Toc70591009)

## Table S1: Summary of MEDLINE and MEDLINE In-Process search terms (via PubMed)

| Criteria | # | Algorithm for MEDLINE and MEDLINE IN PROCESS (via PubMed) | Search hits  (6 June, 2019) |
| --- | --- | --- | --- |
| Population | 1 | Search ("Churg-Strauss Syndrome"[MeSH] OR “eosinophilic granulomatosis with polyangiitis”[TIAB] OR egpa[TIAB] OR “churg strauss”[TIAB] OR “allergic angiitis”[TIAB] OR “allergic granulomatosis”[TIAB] OR “granulomatous small vessel vasculitis”[TIAB] OR “systemic necrotizing vasculitis”[TIAB] OR “systemic necrotising vasculitis”[TIAB] OR (“eosinophilic granulomatosis”[TIAB] AND “polyangiitis”[TIAB]) OR “eosinophilic granulomatous vasculitis”[TIAB]) | 3,388 |
| Study design | 2 | Search ("Epidemiologic Studies"[MeSH] OR “Epidemiologic Measurements"[MeSH] OR epidemiolog*[TIAB] OR "Case-Control Studies"[MeSH] "Retrospective Studies"[MeSH] OR "Cohort Studies"[MeSH] OR prospective[TIAB] OR retrospective[TIAB] OR “cross sectional”[TIAB] OR “trans sectional”[TIAB] OR transversal[TIAB] OR observational[TIAB] OR longitudinal[TIAB] OR “population based”[TIAB] OR naturalistic[TIAB] OR cohort[TIAB] OR “case control”[TIAB] OR “real world evidence”[TIAB] OR "Population Surveillance"[MeSH]) | 2,902,950 |
| Outcomes | 3 | Search ("comorbidity"[MeSH] OR “comorbidity”[TIAB] OR "morbidity"[MeSH] OR “morbidity”[TIAB] OR “incidence”[MeSH] OR incidence[TIAB] OR “prevalence”[MeSH] OR prevalence[TIAB] OR “disease frequency”[TIAB] OR prognosis[TIAB] OR trend[TIAB] OR pattern[TIAB] OR relaps*[TIAB] OR refractory[TIAB] OR asthma[TIAB] OR (nasal[TIAB] AND polyp*[TIAB])) | 3,124,079 |
| Combined | 4 | #2 AND #3 | 924,046 |
| Outcomes (HCRU-specific) | 5 | Search (“healthcare resource” OR “healthcare resources” OR “medical resource”[TIAB] OR “medical resources”[TIAB] OR “healthcare resource use”[TIAB] OR “health resource consumption”[TIAB] OR “health care consumption”[TIAB] OR “medical resource consumption”[TIAB] OR “hospitalization” OR “hospital admission”[TIAB] OR ”hospital admissions”[TIAB] OR “icu admission”[TIAB] OR “emergency department visit”[TIAB] OR “emergency department visits”[TIAB] OR “emergency room visit”[TIAB] OR “emergency room visits”[TIAB] OR “er visit”[TIAB] OR “er visits”[TIAB] OR “ed visit”[TIAB] OR “ed visits”[TIAB] OR “inpatient visit”[TIAB] OR “inpatient visits”[TIAB] OR “outpatient visit”[TIAB] OR “outpatient visits”[TIAB] OR “specialist visit”[TIAB] OR “specialist visits”[TIAB] OR “unscheduled doctor visit”[TIAB] OR “unscheduled doctor visits”[TIAB] OR “unscheduled physician visit”[TIAB] OR “unscheduled physician visits”[TIAB] OR “general practitioner visit”[TIAB] OR “general practitioner visits”[TIAB] OR “gp visit”[TIAB] OR “gp visits”[TIAB] OR “resource use”[TIAB] OR “resource utilisation”[TIAB] OR “resource utilization”[TIAB] OR “health care resource”[TIAB] OR “health care resources”[TIAB] OR “health resource”[TIAB] OR “health resources”[TIAB] OR inpatient[TIAB] OR inpatients[TIAB] OR outpatient[TIAB] OR outpatients[TIAB] OR hcru[TIAB] OR "utilization"[Subheading] OR “Patient Acceptance of Health Care”[MeSH] OR “Hospitalization”[MeSH]) | 662,150 |
| Limits | 6 | Search (#4 OR #5) | 1,483,254 |
|  | 7 | Search (#1 AND #6) | 354 |
|  | 8 | Search ("Animals"[MeSH] NOT "Humans"[MeSH]) | 4,587,309 |
|  | 9 | Search (#7 NOT #8) | 354 |
|  | 10 | Search ("letter"[PT] OR "editorial"[PT] OR "Case Reports" [PT]) | 3,339,791 |
|  | 11 | Search (#9 NOT #10) | 296 |

HCRU, health care resource utilisation; TIAB, title and abstract.

## Table S2: Summary of EMBASE search terms

| **Criteria** | **#** | **Algorithm for Embase (via Embase.com)** | **Search hits  (6 June, 2019)** |
| --- | --- | --- | --- |
| **Population** | 1 | 'eosinophilic granulomatosis with polyangiitis'/exp OR 'eosinophilic granulomatosis with polyangiitis':ti,ab OR 'egpa':ti,ab OR 'churg strauss':ti,ab OR 'allergic angiitis':ti,ab OR 'allergic granulomatosis':ti,ab OR 'granulomatous small vessel vasculitis':ti,ab OR 'systemic necrotizing vasculitis':ti,ab OR 'systemic necrotising vasculitis':ti,ab | 4,510 |
| **Study design** | 2 | 'epidemiology'/exp OR 'epidemiological data'/exp OR epidemiolog*:ti,ab OR 'case control study'/exp OR 'retrospective study'/exp OR 'cohort analysis'/exp OR 'longitudinal study'/exp OR 'follow up'/exp OR 'prospective study'/exp OR 'cross-sectional study'/exp OR 'trend study'/exp OR prospective:ti,ab OR retrospective:ti,ab OR 'cross sectional':ti,ab OR 'trans sectional':ti,ab OR transversal:ti,ab OR observational:ti,ab OR longitudinal:ti,ab OR 'population based':ti,ab OR naturalistic:ti,ab OR cohort:ti,ab OR 'case control':ti,ab OR 'real world evidence':ti,ab OR 'health survey'/exp OR 'surveillance':ti,ab | 6,689,936 |
| **Outcomes** | 3 | 'comorbidity'/exp OR 'comorbidity':ti,ab OR 'morbidity'/exp OR 'morbidity':ti,ab OR 'incidence'/exp OR 'incidence':ti,ab OR 'prevalence'/exp OR 'prevalence':ti,ab OR 'disease frequency':ti,ab OR 'surveillance':ti,ab OR 'health survey'/exp OR 'prognosis':ti,ab OR 'trend':ab,ti OR 'pattern':ab,ti OR relaps*:ti,ab OR 'refractory':ti,ab OR 'asthma':ti,ab OR ('nasal':ti,ab AND polyp*:ti,ab) | 4,737,821 |
| **Study design + outcomes** | 4 | #2 AND #3 | 2,884,169 |
| **Outcomes (HCRU-specific)** | 5 | 'healthcare resource' OR 'healthcare resources' OR 'medical resource':ab,ti OR 'medical resources':ab,ti OR 'healthcare utilisation'/exp OR 'health care utilization'/exp OR 'healthcare use'/exp OR 'healthcare resource use':ab,ti OR 'health resource consumption':ab,ti OR 'health care consumption':ab,ti OR 'medical resource consumption':ab,ti OR 'hospitalisation' OR 'hospitalization'/exp OR 'hospital admission':ab,ti OR 'hospital admissions':ab,ti OR 'icu admission':ab,ti OR 'emergency department visit':ab,ti OR 'emergency department visits':ab,ti OR 'emergency room visit':ab,ti OR 'emergency room visits':ab,ti OR 'er visit':ab,ti OR 'er visits':ab,ti OR 'ed visit':ab,ti OR 'ed visits':ab,ti OR 'inpatient visit':ab,ti OR 'inpatient visits':ab,ti OR 'outpatient visit':ab,ti OR 'outpatient visits':ab,ti OR 'specialist visit':ab,ti OR 'specialist visits':ab,ti OR 'unscheduled doctor visit':ab,ti OR 'unscheduled doctor visits':ab,ti OR 'unscheduled physician visit':ab,ti OR 'unscheduled physician visits':ab,ti OR 'general practitioner visit':ab,ti OR 'general practitioner visits':ab,ti OR 'gp visit':ab,ti OR 'gp visits':ab,ti OR 'resource use':ab,ti OR 'resource utilisation':ab,ti OR 'resource utilization':ab,ti OR 'health care resource':ab,ti OR 'health care resources':ab,ti OR 'health resource':ab,ti OR 'health resources':ab,ti OR inpatient*:ab,ti OR outpatient*:ab,ti OR hcru:ab,ti | 814,089 |
| **Population + Study design + Outcomes (all)** | 6 | #1 AND (#4 or #5) | 1,231 |
| **Limits** | 7 | [animals]/lim NOT [humans]/lim | 5,596,412 |
|  | 8 | #6 NOT #7 | 1,223 |
|  | 9 | letter:it OR editorial:it OR 'case report'/exp | 3,883,023 |
|  | 10 | #8 NOT #9 | 964 |
| **ERS, ATS, EULAR, and ACR Conference Abstract Searches** | 11 | 'annals of the rheumatic diseases'/jt AND ([conference abstract]/lim OR [conference paper]/lim OR [conference review]/lim) | 22,595 |
|  | 12 | 'arthritis and rheumatology'/jt AND ([conference abstract]/lim OR [conference paper]/lim OR [conference review]/lim) | 12,419 |
|  | 13 | 'european respiratory journal'/jt AND ([conference abstract]/lim OR [conference paper]/lim OR [conference review]/lim) | 31,093 |
|  | 14 | 'american journal of respiratory and critical care medicine'/jt AND ([conference abstract]/lim OR [conference paper]/lim OR [conference review]/lim) | 47,078 |
|  | 15 | #11 OR #12 OR #13 OR #14 | 113,185 |
| **Combined** | 16 | #6 AND #15 | 191 |
|  | 17 | #10 OR #16 | 989 |

ACR, American College of Rheumatology; ATS, American Thoracic Society; ERS, European Respiratory Society EULAR, European League Against Rheumatology HCRU, healthcare resource use.

## Table S3: PICOS eligibility criteria

| **Category** | **Inclusion criteria** | **Exclusion criteria** |
| --- | --- | --- |
| **Population** | Patients aged ≥18 years with EGPA (previously referred to as CSS) identified based on the following diagnostic criteria or validated algorithms:   - ACR 1990 - CHCC 1994/2012 - Lanham 1984 - EMEA 2007 - ICD-9* 446.4 and ICD-10 M30.1 | Studies with unclear reporting on diagnostic criteria or unclear validated algorithms |
| **Interventions/comparators**^†^ | N/A | N/A |
| **Outcomes** | **Co-primary outcomes**   - Annual incidence and prevalence   **Exploratory outcomes**   - Morbidity: - EGPA relapse/refractory disease - Comorbidities: severe asthma and/or nasal polyps - HCRU: - Hospitalisation events - ED visits - Specialist/GP visits | Any other outcome |
| **Study Design** | Observational, real-world studies (prospective and retrospective cohort studies, cross-sectional, case-control studies) | Experimental studies (RCT, non-RCT, single-arm trials) |
| **Other** | All years up to 6 June, 2019 (with no lower limit on date)  Publications reported in the English language | Publications reported in languages other than English |

*ICD-9 code 446.4 identifies EGPA and granulomatosis with polyangiitis, without distinguishing between the conditions. This issue was addressed by the ICD-10 system, which has a specific code for EGPA (M30.1); ^†^The systematic literature review was conducted in population-based, real-world studies aimed at investigating the incidence, prevalence, and morbidity associated with EGPA; it did not explore the effectiveness of treatment options.

ACR, American College of Rheumatology; CHCC, Chapel Hill consensus conference; CSS, Churg-Strauss syndrome; ED, emergency department; EGPA, eosinophilic granulomatosis with polyangiitis; EMEA, European Medicines Evaluation Agency; GP, general practitioner; HCRU, healthcare resource utilisation; ICD-9, International Classification of Diseases, Ninth Revision; ICD-10, International Classification of Diseases, 10^th^ Revision; N/A, not applicable; RCT, randomized controlled trial.

## Table S4: Summary of included incidence and prevalence studies

| Source  (Author, Year) | Study Design | Country | Study Time Period | Diagnostic Criteria /Nomenclature Used | Data Source | Brief Target Population Description |
| --- | --- | --- | --- | --- | --- | --- |
| Wójcik, 2018 [1] | Multicenter, retrospective | Poland | Jan 1990 to Dec 2016 | CHCC 2012 | Scientific Consortium of the Polish Vasculitis Registry (POLVAS) | Adult patients diagnosed with AAV |
| Bell, 2018 [2] | Retrospective study | US | Database # 1:  1 Oct, 2015–31 Dec, 2016  Database # 2:  1 Oct, 2015–31 Mar, 2017 | ICD-10 | Two administrative claims databases | Patients with EGPA identified using ICD-10 codes |
| Gokhale, 2018 [3] | Retrospective, database | US | 2010 to 2014 | NR | US commercial claims database | Adult patients with asthma |
| Nilsen, 2018 [4] | Retrospective, database | Norway | 1999 to 2013 | EMEA 2007 | 11 hospital databases | Adult patients diagnosed with AAV |
| Herlyn, 2017 [5] | Population-based prospective study | Germany | 1 Jan, 1998 and 31 Dec, 2014 | CHCC 1994 | (a) departments of all hospitals, including their outpatient clinics; (b) all departments of pathology, and (c) the reference immunological laboratories serving the catchment area | All newly diagnosed cases of AAV (GPA, MPA, EGPA) |
| Rodriguez-Muguruza, 2016 [6] | Retrospective | Spain | 1984–2015 | NR | Hospital database of all muscular biopsies performed | Patients with systemic vasculitis |
| Pamuk, 2013 [7] | Retrospective | Turkey | NR: within the last 10 years | NR | The hospital medical center in Edirne (the only tertiary referral center for rheumatic diseases for a mixed rural and urban population) | Patients (≥16 years) with ANCA-AAV |
| Fujimoto, 2011 [8] | Population-based prospective study | Japan, UK | 1 Jan, 2005–31 Dec, 2009 | EMEA 2007 | Five renal, three rheumatology and two otolaryngology units/referral centers in Japan. Norfolk and Norwich University Hospital in UK | Patients with new-onset cases of EGPA identified through the referral centres |
| Berti, 2017 [9] | Population-based retrospective study | US | Incident cohort: 1 Jan, 1996–31 Dec, 2015  Prevalence cohort: 1 Jan, 2015 | ACR 1990; CHCC 2012; EMEA 2007 | Rochester Epidemiology Project (REP), a medical records linkage system containing records from all the medical providers of the local population | AAV cases with at least 1 diagnosis code or at least 1 positive laboratory test (ANCA) |
| Kanecki, 2017 [10] | Population-based retrospective study | Poland | 2008–2013 | NR | Polish National Institute of Public Health survey | All first-time hospitalised EGPA patients from all hospitals, except psychiatric and military patients. |
| Pamuk, 2016 [11] | Population-based retrospective study | Turkey | Dec 2004–Dec 2014 | ACR 1990; CHCC 1994 | Trakya University Medical Facility in Edirne (Thrace, Turkey) | Patients were identified from hospital records and from ANCA serology databases. ICD-10 to identify EGPA patients. |
| Nesher, 2016 [12] | Population-based retrospective study | Israel | 1990–2009 | ACR 1990;  CHCC 1994 | Two major medical centers, Hadassah and Shaare-Zedek in Jerusalem | Patients with EGPA identified through the medical records of two medical centres. |
| Romero-Gomez, 2015 [13] | Population-based retrospective study | Spain | 1994–2010 | ACR 1990; CHCC 2012 | Costa del Sol Hospital in Marbella, a secondary hospital and two tertiary hospitals, Hospiten Clinic in Estepona and USP Hospital in Marbella | Patients aged 14 years or older diagnosed with vasculitis located by automated database analysis using ICD-9 447.6 for EGPA |
| Jaffe, 2014 [14] | Retrospective | Australasia | Jul 2009–Jul 2013 | NR | Cases were reported electronically by Australian TSANZ members to ARNOLD | Patients with rare orphan lung diseases |
| Sada, 2014 [15] | Population-based cross-sectional study | Japan | 2008 | ACR 1990; Lanham criteria 1984 | Department of internal medicine, rheumatology, and neurology at the 2,599 hospitals, which were randomly selected from all hospitals in Japan | NR (The survey is sent to hospitals asking only for the number of patients with EGPA treated during 2008) |
| Herlyn, 2014 [16] | Population-based retrospective study | Germany | 1 Jan–31 Dec, 2006 | EMEA 2007 | The hospital departments where the authors are employed (Department of Rheumatology, University Medical Centre Schleswig-Holstein, Campus Luebeck and the Department of Rheumatology of the Klinikum Bad Bramstedt); (ii) all departments of the University Hospital of Schleswig-Holstein, Campus Luebeck, including their outpatient clinics; (iii) all other hospitals; (iv) all departments of pathology; (v) all physicians in private practice; (vi) pension funds; (vii) health insurance providers; (viii) death registries and (ix) the reference immunology laboratories serving the catchment area. An additional data source was the Vasculitis Register Schleswig-Holstein. | Patients with EGPA with ICD code of M30.3 |
| Jaffe, 2012 [17] | Retrospective | Australasia | Jul 2009–Jul 2013 | NR | Cases were reported electronically by Australian TSANZ members to ARNOLD | Patients with rare orphan lung diseases |
| Mohammad, 2011 [18] | Population-based prospective study | Sweden | 1 Jan, 2010 | EMEA 2007 | Hospital records and a serology ANCA database. | Patients with AAV and PAN |
| Vinit, 2011 [19] | Population-based retrospective study | France | Jan 1998–May 2008 | ACR 1990 | Hospitalisation data in Burgundy | Patients with EGPA diagnosed using ICD-10, identified using the specific EGPA code from the hospitalisation summaries in Burgundy |
| Mohammad, 2009 [20] | Population-based prospective study | Sweden | 1 Jan 1997–31 Dec, 2006 | ACR 1990; CHCC 1994 | Clinical databases at the Departments of Nephrology and Rheumatology at the University Hospitals of Lund and Malmö and the Department of Internal Medicine at the Hospitals of Landskrona and Trelleborg | All newly diagnosed cases, who fulfilled the study criteria of EGPA. ICD-10 codes were used to identify cases |
| Watts, 2009 [21] | Retrospective | UK | 1988–2007 | ACR 1990;  CHCC 1994 | NORVASC registry | Patients with ANCA-AAV |
| Ormerod, 2008 [22] | Population-based retrospective study | Australia | 1 Jan, 1995–31 Dec, 2004 | ACR 1990 | Clinical databases of inpatients at two teaching hospitals within the ACT service that includes the entire ACT, as well as SE-NSW. | Patients fulfilling the World Health Organization International Classification of Disease (ICD) (versions 9 and 10) codes |
| Herlyn, 2008 [23] | Population-based prospective study | Germany | 1 Jan, 1998–31 Dec, 2005 | CHCC 1994 | All hospital departments in the catchment area, including outpatient clinics; all departments of pathology; and the reference immunological laboratories serving the catchment area | All new cases of EGPA from 1 Jan, 1998. |
| Mohammad, 2007 [24] | Population-based cross-sectional study | Sweden | 2003 | ACR 1990 | Departments and hospitals inside the study area: (i) Lund University Hospital: the databases for the Departments of Internal Medicine (including Nephrology, Pulmonology and General Internal Medicine), Rheumatology, ENT, Ophthalmology, Dermatology, Infectious diseases and Paediatrics; (ii) Landskrona Hospital: the databases for the Departments of Medicine; and (iii) the largest private out-patient clinic and two arbitrarily chosen primary healthcare centres. Outside the study area the following databases were utilised: (i) Malmö University Hospital: the databases for the Departments of Internal Medicine (including Rheumatology and Nephrology sections); (ii) Helsingborg Hospital: Department of Internal Medicine; and (iii) Trelleborg Hospital: Department of Medicine. | Patients with EGPA identified through clinical records, including hospital discharge records and databases listing out-patient clinics, using the I ICD-10 codes |
| Mahr, 2004 [25] | Population-based cross-sectional study | France | 1 Jan–31 Dec, 2000 | ACR 1990 | Survey to general practitioners, the departments of all the public hospitals and 2 large private clinics, and the National Health Insurance System | Patients with known cases of EGPA |
| Gonzalez-Gay, 2003 [26] | Population-based retrospective study | Spain | Jan 1988–Dec 2001 | CHCC 1994 | Department of medicine of the Hospital Xeral-Calde | Patients with a new diagnosis of EGPA |
| Watts, 2001 [27] | Population-based prospective study | UK, Spain | 1 Jan, 1988–31 Dec, 1998 | ACR 1990;  CHCC 1994 | Hospital based in two regions of Europe (UK and Spain) | Patients (> 15 years) with new onset primary systemic vasculitis |
| Watts, 2000[28] | Population-based prospective study | UK | 1 Jan, 1988–31 Dec, 1997 | ACR 1990 | Hospital based in the former Norwich Health Authority, Norfolk, UK | Patients (outpatient or inpatient and registered with a general practitioner) with new clinical diagnosis of systemic vasculitis |
| Martin, 1999 [29] | Population-based retrospective study | UK | Sept 1984–Jun 1996 | NR | Prescription-event monitoring (PEM) database of the Drug Safety Research Unit | The data were collected as a part of 58 PEM studies, in which a questionnaire was sent to the prescriber asking for ‘any new diagnosis, any reason for referral to a consultant or admission to hospital, any unexpected deterioration (or improvement) in a concurrent illness, any suspected adverse drug reaction, or any other complaint which was considered of sufficient importance to enter in the patient's notes'. Events that had been coded as EGPA/CSS were identified |
| Haugeberg, 1998 [30] | Population-based retrospective study | Norway | Jan 1992–Dec 1996 | ACR 1990 | Norwegian community hospital | Patients with EGPA identified through ICD-9 codes |
| Watts, 1995 [31] | Population-based retrospective study | UK | 1 Feb, 1988–31 Jan, 1994 | ACR 1990;  Lanham criteria 1984 | Hospital-based in the Norwich Health Authority, Norfolk, UK | Patients attending with a new diagnosis of systemic vasculitis |
| Pearce, 2016 [32] | Retrospective study | UK | 1 Mar, 2007–30 Jun, 2013 | EMEA 2007 | Hospital trust in Nottingham from the following five sources: rheumatology departmental register; renal departmental database search; positive MPO/PR3 ANCA results; histopathology laboratory database of biopsies coded for vasculitis; and inpatient discharges coded as vasculitis. patients at the hospital in Derby were identified from the following three sources: rheumatology department database search; renal departmental database search; and positive MPO/PR3 ANCA results | Newly diagnosed ANCA-associated vasculitis |
| Reinhold-Keller, 2002 [33] | Population-based prospective study | Germany | 1 Jan, 1988–31 Dec, 1999 | ACR 1990; CHCC 1994 | (i) all departments of all hospitals including their out‐patient clinics, and including the departments at which the authors are employed; (ii) all departments of pathology; and (iii) all reference immunology laboratories serving the respective catchment areas | All newly diagnosed cases of EGPA |
| Dadoniene, 2005 [34] | Population-based retrospective study | Lithuania | 1990–1999 | ACR 1990 | Tertiary nephrology, dermatology, and internal medicine departments of Vilnius hospital, along with pathology data and renal registers. Patients referred to Vilnius University Hospital rheumatology department were prospectively included in the study. | Patients diagnosed with EGPA in the 10 year period from 1990 to 1999 in Vilnius. |
| Reinhold-Keller, 2000 [35] | Population-based cross-sectional study | Germany | 1994 | CHCC 1994 | (A) the departments at which the authors are employed: the Department of Rheumatology, University of Lübeck, the Rheumaklinik Bad Bramstedt GmbH (Segeberg), the Department of Rheumatology and Clinical Immunology, University of Freiburg, including their outpatient clinics; (B) all departments of the medical universities, all other hospitals, including their outpatient clinics serving the respective catchment areas; (C) all departments of pathology at both participating universities and all participating hospitals, plus pathologists in private practice; (D) all physicians in private practice; (E) pension funds; (F) health insurance providers; (G) central death registries; and (H) the reference immunology laboratories of both universities serving the respective catchment areas. | Patients with EGPA between 1 Jan and 31 Dec, 1994, in the catchment area |

AAV; ANCA-associated vasculitides; ACR, American College of Rheumatology; ACT, Australian Capital Territory; ANCA, anti-neutrophil cytoplasmic antibody; ARNOLD, Australasian Registry Network for Orphan Lung Disease; CHCC, Chapel Hill Consensus Conference; CSS, Churg–Strauss syndrome; DB, database; EGPA, eosinophilic granulomatosis with polyangiitis; EMEA, European Medicines Agency; ENT, ear, nose and throat; GPA, granulomatosis with polyangiitis; ICD, International Classification of Diseases; MPA, microscopic polyangiitis; MPO, myeloperoxidase; NHA, Norwich Health Authority; PAN, polyarteritis nodosa; PR3, proteinase 3; SE-NSW, Southeastern New South Wales; TSANZ, Thoracic Society of Australia and New Zealand; UK, United Kingdom; US, United States.

## Table S5: Summary of included morbidity and HCRU studies

| Author, year | Study Design | Country | Patient Accrual Years | Diagnostic Criteria Used | Study population definition | EGPA Patients (N) | EGPA population definition | Follow-up Time |
| --- | --- | --- | --- | --- | --- | --- | --- | --- |
| Whyte, 2011 [36] | Retrospective chart review | Australia | 2002–2008 | NR | Patients with CSS | 19 | 9 p-ANCA/MPO+, 2 c-ANCA/PR3+, 8 ANCA negative | NR |
| Haq, 2013 [37] | Retrospective chart review | Bangladesh | May 2013–Jul 2013 | NR | Patients receiving treatment for vasculitis | 2 | Patients with EGPA receiving treatment | NR |
| Kawano-Dourado, 2017 [38] | Retrospective study | Brazil | 2012–2016 | NR | Patients with the diagnosis of GPA or EGPA that needed rituximab therapy | 10 | Patients with the diagnosis of EGPA that needed rituximab therapy | NR |
| Pulenzas, 2018 [39] | Retrospective study | Canada | NR | NR | Patients with EGPA diagnosis | 25 | Patients with EGPA diagnosis | 12 months |
| Pulenzas, 2018 [40] | Retrospective chart review | Canada | 1967–2017 | NR | Patients diagnosed with EGPA | 110 | Patients with EGPA diagnosis | Mean (SD): 99.2 (102.8) months |
| Cottin, 2016 [41] | Retrospective multicenter study | France | NR | 94%: ACR criteria; 6% all had at least two extra-thoracic organ manifestations attributable to the disease | Patients with EGPA | 157 | Patients with EGPA | Mean (SD): 7.4 years (6.4) |
| Comarmond, 2013 [42] | Retrospective cohort study | France | 1957–2009 | ACR 1990; CHCC | Patients diagnosed with EGPA | 383 | Patients diagnosed with EGPA | Mean (SD): 66.8 (62.5) months |
| Pagnoux, 2011 [43] | Retrospective cohort study | France | 1957–2009 | ACR 1990; CHCC | Patients diagnosed with CSS | 383 | Patients diagnosed with CSS | Mean (SD): 66.8 (62.5) |
| Guilpain, 2009 [44] | Retrospective cohort study | France | NR | ACR 1990; CHCC | Vasculitis patients with anti-MPO antibodies | 53 | Patients with CSS | NR |
| Vinit, 2011 [19] | Retrospective study | France | 1998–2008 | ACR 1990 | Patients with CSS | 31 | ANCA positive (26%) | Mean: 7.7 years Median: 5 years |
| Terrier, 2009 [45] | Retrospective study | France | Jan 1996–Dec 2006 | ACR 1990 | Patients with anti-MPO-associated vasculitides | 5 | Patients with CSS | Mean (SD): 54 months (38) |
| Guillevin, 1999 [46] | Prospective/retrospective study | France | 1963–1995 | ACR 1990 | Patients with CSS | 96 | Patients with CSS | NR |
| Hot, 2013 [47] | Retrospective/prospective multicenter survey | France, UK, US | NR | NR | Patients with EGPA with relapsing or refractory disease treated with rituximab | 30 | Patients with EGPA with relapsing or refractory disease treated with rituximab | Median: 40 months |
| Seeliger, 2017 [48] | Retrospective cohort study | Germany | 1999–2015 | ACR 1990 | Patients diagnosed EGPA per ACR criteria | 30 | Patients diagnosed EGPA per ACR criteria  ANCA-positive: 1 (3%) | Median: 31 (10-39) months |
| Mahrhold, 2018 [49] | Retrospective study | Germany | 2008–2017 | ACR 1990; CHCC 1994 | Patients with EGPA | 55 | Patients with EGPA  ANCA-negative: 89.1% MPO-ANCA positive: 9.1% PR3-ANCA positive: 1.8% | Median (SD): 33.2 months |
| Yathish, 2014 [50] | Retrospective study | India | 2003–2014 | NR | Patients with CSS | 28 | Patients with CSS  ANCA-positive: 78.57% | Mean (SD): 46.9 months (29.3) |
| Latorre, 2013 [51] | Cross-sectional study | Italy | NR | NR | Patients with EGPA  ANCA-positive: 12 (37%) | 32 | Patients with EGPA  ANCA-positive: 12 (37%) | NR |
| Bacciu, 2008 [52] | Retrospective study | Italy | 1997–2004 | Lanham’s criteria; ACR 1990; CHCC 1994 | Patients diagnosed with CSS | 17 | Patients diagnosed with CSS and nasal polyposis.  ANCA positive: 8 (47%) ANCA negative: 9 (53%) | Minimum of 12 months |
| Bacciu, 2006 [53] | Retrospective study | Italy | Jan 1997–Jan 2004 | ACR 1990 | Patients with CSS | 21 | Patients with CSS with ENT involvement | Mean (SD): 43.7 months (19.9) Range: 13 to 87 months |
| Seccia, 2018 [54] | Cross-sectional study | Italy | 2010–2014 | ACR 1990 | Patients with EGPA diagnosis | 39 | Patients with EGPA diagnosis | NR |
| Bottero, 2007 [55] | Retrospective chart review | Italy | 1985–2005 | CHCC | Patients with CSS | 51 | Patients with CSS. Allergic ANCA -positive (n=3) Allergic ANCA-negative (n=8)  Non-allergic ANCA-positive (n=11) Non-allergic ANCA-negative (n=20) | NR |
| Pavone, 2006 [56] | Retrospective chart review | Italy | 1985–2003 | ACR | Patients with primary small vessel vasculitides | 23 | Patients with CSS ANCA evaluable (n=22) ANCA negative: 55% cANCA: 9% Anti-PR3: 5% pANCA: 36% Anti-MPO: 32% | NR |
| Latorre, 2015 [57] | Cross-sectional study | Italy | NR | NR | Patients with EGPA and CSA | 40 | Patients with EGPA diagnosis | NR |
| Latorre, 2012 [58] | Cross-sectional study | Italy | NR | NR | Patients diagnosed with CSS | 35 | Patients diagnosed with CSS | NR |
| Latorre, 2012 [59] | Cross-sectional study | Italy | NR | ACR | 20 consecutive patients with a diagnosis of CSS | 20 | 20 consecutive patients with a diagnosis of CSS | NR |
| Latorre, 2013 [60] | Cross-sectional study | Italy | NR | NR | Patients diagnosed with EGPA | 36 | Patients diagnosed with EGPA | NR |
| Baldini, 2013 [61] | Retrospective chart review | Italy | 1989–2011 | ACR 1990 | Patients diagnosed with CSS | 48 | Patients diagnosed with CSS | Mean (SD): 7.2 (5.3) years |
| Baldini, 2011 [62] | Cross-sectional/prospective study | Italy | NR | ACR 1990 | Consecutive patients with a diagnosis of CSS (ACR criteria) | 20 | Patients diagnosed with CSS | NR |
| Latorre, 2013 [63] | Cross-sectional study | Italy | NR | NR | Patients diagnosed with EGPA | 32 | Patients diagnosed with EGPA | Mean (SD): 6 (5) years |
| Baldini, 2013 [64] | Retrospective cohort study | Italy | NR | ACR 1990 | Patients diagnosed with EGPA | 47 | Patients diagnosed with EGPA | Mean (SD): 7 (5) years |
| Jeannin, 2013 [65] | Retrospective study | Italy | 2006–2013 | NR | Patients with AAV treated with rituximab | 2 | Patients with CSS treated with rituximab | 7 years |
| Baldini, 2012 [66] | Cross-sectional/prospective study | Italy | NA (cross sectional study) | ACR 1990 | Patients diagnosed with EGPA | 26 | Patients diagnosed with EGPA | Mean (SD: 6 (5) years |
| Watanabe, 2018 [67] | Prospective cohort study | Japan | 2009–2010 and 2011–2014 | EMEA 2007 algorithm | Patients with MPO-AAV | 15 | Patients diagnosed with EGPA | Minimum 6 months and up to 24 months |
| Saku, 2018 [68] | Retrospective study | Japan | 1996–2015 | Lanham's criteria; ACR 1990 | Patients with EGPA diagnosis who met the American College of Rheumatology (ACR) 1990 criteria for the classification of CSS or Lanham’s criteria | 188 | Patients with EGPA diagnosis who met the American College of Rheumatology (ACR) 1990 criteria for the classification of CSS or Lanham’s criteria  MPO-ANCA–positive: 78 (41.9%) PR3-ANCA–positive: 10 (5.4%) | Median: 56 (21–102) months |
| Nakamoto, 2018 [69] | Retrospective chart review | Japan | 2001–2015 | Lanham's criteria; ACR 1990 | Patients with EGPA diagnosis and comorbid bronchial asthma | 31 | Patients with EGPA with comorbid bronchial asthma | NR |
| Tsurikisawa, 2017 [70] | Retrospective study | Japan | 1999–2015 | ACR 1990 | Patients with EGPA diagnosis | 121 | Patients with EGPA diagnosis | 114 (94.2%) were followed for a mean of 8.2 ± 5.8 years after diagnosis; the remaining 7 patients (5.8%) were lost to follow-up by the end of our study |
| Saku, 2017 [71] | Retrospective study | Japan | 1996–2015 | ACR 1990 | Patients diagnosed with EGPA | 188 | Patients diagnosed with EGPA.  ANCA-positive: 44.7% | Median: 56 months |
| Hasegawa, 2015 [72] | Retrospective study | Japan | Jul 2010–Mar 2013 | ICD-10 code, M301 | Patients with EGPA | 2,195 | Patients with EGPA | NR |
| Yoo, 2017 [73] | Retrospective chart review | Korea | Oct 2000–Aug 2016 | ACR 1990 criteria; algorithm for the classification of AAV | Patients with AAV | 30 | Patients with EGPA  MPO-ANCA: 36.7% PR3-ANCA: 10.0% ANCA negative: 53.3% | Mean (SD): 72.5 months (34.6) |
| Lee, 2012 [74] | Retrospective study | Korea | 1995– 2011 | NR | Patients diagnosed with CSS | 47 | Patients diagnosed with CSS | Median (range): 48.6 (3-152) months |
| Haugeberg, 1998 [30] | Retrospective study | Norway | Jan 1992–Dec 1996 | ACR 1990 | Patients with primary vasculitis | 2 | Patients with CSS | NR |
| Sokolowska, 2014 [75] | Retrospective/prospective cohort | Poland | 1998–2006 | ACR 1990 | Patients with EGPA with ANCA measurements | 50 | 15 ANCA-positive patients, 35 ANCA-negative patients | Median time of the entire observation for all patients: 60 [24-96.5] months |
| Sokołowska, 2013 [76] | Retrospective study | Poland | 1998–2011 | ACR 1990 | Patients with CSS | 30 | Patients with CSS | NR |
| Szczeklik, 2011 [77] | Retrospective /prospective study | Poland | Jul 1999–Mar 2010 | ACR 1990 | Patients with CSS | 22 | Patients with a diagnosis of CSS | NR |
| Wójcik, 2019 [78] | Retrospective multicentre study | Poland | 1990–2016 | ACR 1990; CHCC 2012 | Patients with AAV | 102 | Patients with EGPA  ANCA-positive: 47.8% ANCA-negative: 52.2%  cANCA/anti-PR3: 11.1% pANCA/anti-MPO: 31.1% | NR |
| Kim, 2014 [79] | Retrospective chart review | South Korea | 1990–2011 | ACR 1990 | Patients diagnosed with CSS | 52 | Patients diagnosed with CSS | Median: 1,591 (27–6,707) days |
| Solans-Laqué, 2014 [80] | Retrospective-longitudinal multicentre study | Spain | 1995–2012 | NR | Patients with AAV | 87 | Patients with EGPA  ANCA-positive: NR (66%) MPO ANCA: NR (91%) | Mean (SD): 82.5 (75.3) months |
| Solans-Laqué, 2017 [81] | Retrospective study | Spain | Jan 1990–Jan 2014 | ACR 1990; CHCC 2012 | Patients with AAV | 99 | Patients with EGPA  ANCA-positive: 60.6% | Median (IQR): 82 months (100.4) |
| Armengot, 2013 [82] | Retrospective study | Spain | NR | NR | Patients with GPA, CSS, or CIMDL | 2 | Patients with CSS diagnosis | NR |
| Solans, 2001 [83] | Retrospective chart review | Spain | 1977–1999 | ACR | Patients with CSS | 32 | Patients with CSS ANCA-positive: 53.8% MPO-ANCA: 50% c-ANCA: 3.8% | NR |
| Yılmaz, 2015 [84] | Retrospective/prospective study | Turkey | 2004–2012 | ACR 1990 | Patients diagnosed with EGPA | 14 | Patients with CSS | Median (range): 24 [6-96] months |
| Martin-Suarez, 1997 [85] | Retrospective chart review | UK | 1990–1994 | ACR 1990 | Patients with severe progressive connective tissue disease who were given low dose intravenous cyclophosphamide treatment | 3 | Patients diagnosed with CSS | Median: 56 [5-213] months |
| Reid, 1998 [86] | Retrospective study | UK | 1982–1995 | Lanham criteria; ACR 1990; CHCC | Patients with CSS | 23 | Patients with CSS  ANCA-positive: 59% (10/17) | NR |
| Durel, 2016 [87] | Retrospective study | UK, France, Italy | Jan 1990–Dec 2011 | ACR 1990 | Patients with EGPA | 101 | Patients with EGPA  ANCA-positive: 42.6% ANCA-negative: 56.4% | Median: 6 years |
| Rhee, 2017 [88] | Prospective cohort study | US, Canada | NR | NR | Patients with systemic vasculitis: GPA, EGPA, MPA, GCA, TAK and PAN | 95 | Patients with EGPA | NR (however, from Kaplan–Meier figure, it looks like patients were followed for at least 6 years) |
| Rhee, 2016 [89] | Prospective cohort study | North America | NR | NR | Patients with 6 vasculitides were included if they had no relapse between date of diagnosis and enrollment: GPA, EGPA, MPA, GCA, TAK and PAN. | 95 | Patients with EGPA diagnosis | Not clearly reported (but would be at least 6 years per the figure) |
| Keogh, 2003 [90] | Retrospective study | US | 1990–2000 | Lanham’s criteria; ACR 1990; CHCC 1994 | Patients diagnosed with CSS | 91 | Patients diagnosed with CSS | Median: 2.3 (1-5.5) years |
| Berti, 2018 [91] | Retrospective study | US | 1990–2017 | Lanham’s criteria, ACR 1990, CHCC | Patients diagnosed with EGPA | 63 | Patients with EGPA diagnosis  Positive p-ANCA/MPO-ANCA: 35.6% (21/59) | Median: 55 months (19–136) |
| Santos-Pinheiro, 2015 [92] | Retrospective chart review | US | Jan 2000–May 2013 | ACR | Patients with EGPA | 11 | Patients with EGPA | Median: 33 months Range: 10 to 132 months |
| Abu-Shakra, 1994 [93] | Retrospective study | US | 1979–1993 | ACR 1990 | Patients with PAN and CSS | 12 | Patients with CSS | Mean: 5.5 years Range: 0.25 to 15 years |
| Bell, 2018 [94] | Retrospective study (large claims database) | US | Jul 2007–Mar 2017 | Published algorithms (pre-ICD-10 implementation) and ICD-10 (post Oct 2015) | Patients with EGPA | 2,226 | Patients with EGPA | NR |
| Bell, 2018 [2] | Retrospective study (large claims database) | US | Oct 2015–Mar 2017 | ICD-10 diagnosis codes | Patients with EGPA | 567* 413^†^ | Patients with EGPA | NR |
| Denis, 2017 [95] | Retrospective nationwide study | NR | NR | ACR 1990; CHCC | Patients with EGPA meeting the ACR/CHCC criteria | 33 | Patients with EGPA meeting the ACR/CHCC criteria | Median: 20 months |
| Zagvozdkina, 2017 [96] | Prospective cohort study | NR | NR | ACR 1990; CHCC 2012 | Patients with EGPA meeting the ACR and CHCC criteria  ANCA-positive: 37 (39.8%) | 93 | Patients with EGPA meeting the ACR and CHCC criteria  ANCA-positive: 37 (39.8%) | Mean (SD): 6.3 (6.5) years or 587.7 patient-years |
| Seeliger, 2014 [97] | Retrospective cohort study | NR | NR | ACR | Patients with EGPA defined per ACR criteria | 25 | Patients with EGPA defined per ACR criteria | NR |
| Zagvozdkina, 2017 [98] | Prospective study | NR | NR | ACR 1990; CHCC 2012 | Patients with EGPA | 93 | Patients with EGPA | Mean (SD): 6.3 years (6.5) |
| Zagvozdkina, 2017 [99] | Prospective study | NR | NR | ARC 1990; CHCC 2012 | Patients with EGPA | 71 | Patients with EGPA  ANCA-positive: 52.1% | Mean (SD): 4.5 years (3.3) |
| Grayson, 2015 [100] | Prospective cohort study | NR | 2006–2013 | ACR 1990 | Patients with EGPA | 141 | Patients with EGPA  ANCA-positive: 46% ANCA-negative: 54% | NR |

*Number of patients in the Truven MarketScan Commercial Claims and Encounters database; ^†^number of patients in the Optum Clinformatics Data Mart database.
AAV, ANCA-associated vasculitis; ACR, American College of Rheumatology; ANCA, anti-neutrophil cytoplasmic antibodies; CHCC, Chapel Hill consensus conference; CIMDL, cocaine-induced midline destructive lesions; CSA, chronic severe asthma; CSS, Churg–Strauss syndrome; EGPA, eosinophilic granulomatosis with polyangiitis; ENT, ear, nose, throat; GCA, giant cell arteritis; GPA, granulomatosis with polyangiitis; HCRU, healthcare resource utilisation; ICD, International Classification of Diseases; MPA, microscopic polyangiitis; MPO, myeloperoxidase; NA, not applicable; NR, not reported; PAN, polyarteritis nodosa; PR3, proteinase 3; SD, standard deviation; TAK, Takayasu’s arteritis; UK, United Kingdom; US, United States.

## Table S6: Risk of bias quality assessment for incidence and prevalence studies

| Author, year | 1. Was the sample representative of the target population? | 2. Were study participants recruited in an appropriate way? | 3. Was the sample size adequate? | 4. Were the study subjects and the setting described in detail? | 5. Was the data analysis conducted with sufficient coverage of the identified sample? | 6. Were objective, standard criteria used for the measurement of the condition? | 7. Was the condition measured reliably? | 8. Was there appropriate statistical analysis? | 9. Are all important confounding factors/subgroups/differences identified and accounted for?  (Y / N / UC / NA) | 10. Were subpopulations identified using objective criteria? |
| --- | --- | --- | --- | --- | --- | --- | --- | --- | --- | --- |
| Reinhold-Keller, 2005 | Y | Y | Y | N | Y | Y | Y | Y | N | NA |
| Watts, 2001 | N | Y | N | N | Y | Y | Y | Y | N | NA |
| Watts, 2000 | N | Y | N | N | Y | Y | Y | Y | N | NA |
| Fujimoto, 2011 | N | Y | N | N | Y | Y | Y | UC | N | NA |
| Berti, 2017 | Y | Y | N | Y | Y | Y | Y | Y | Y | Y |
| Kanecki, 2017 | Y | Y | Y | Y | Y | N | N | Y | N | NA |
| Pamuk, 2016 | N | Y | N | N | Y | Y | Y | Y | N | NA |
| Nesher, 2016 | N | Y | N | Y | Y | Y | Y | UC | N | NA |
| Romero-Gomez, 2015 | N | Y | N | Y | Y | Y | Y | Y | N | NA |
| Sada, 2014 | Y | Y | Y | Y | Y | UC | UC | N | N | NA |
| Herlyn, 2014 | Y | Y | Y | Y | Y | Y | Y | Y | N | Y |
| Vinit, 2011 | Y | Y | Y | Y | Y | Y | Y | N | N | NA |
| Mohammad, 2009 | N | Y | N | Y | Y | Y | Y | N | N | NA |
| Ormerod, 2008 | N | Y | N | Y | Y | Y | Y | Y | N | NA |
| Herlyn, 2008 | Y | Y | N | Y | Y | Y | Y | Y | N | NA |
| Mohammad, 2007 | Y | Y | N | Y | Y | Y | Y | Y | N | NA |
| Mahr, 2004 | N | Y | N | Y | Y | Y | Y | Y | N | NA |
| Gonzalez-Gay, 2003 | N | Y | N | N | Y | Y | Y | Y | Y | Y |
| Reinhold-Keller, 2002 | Y | Y | N | Y | Y | Y | Y | Y | N | NA |
| Martin, 1999 | UC | Y | N | UC | Y | UC | UC | Y | N | NA |
| Haugeberg, 1998 | N | Y | N | UC | Y | Y | Y | N | N | NA |
| Watts, 1995 | N | Y | N | N | Y | Y | Y | N | N | NA |
| Reinhold-Keller, 2000 | Y | Y | N | Y | Y | Y | Y | Y | N | NA |
| Dadoniene, 2005 | N | Y | N | UC | Y | Y | Y | N | N | NA |
| Bell, 2018 | UC | Y | Y | UC | UC | Y | UC | UC | UC | NA |
| Herlyn, 2017 | Y | Y | Y | N | Y | Y | Y | UC | UC | NA |
| Mohammad, 2011 | N | Y | N | N | Y | Y | Y | Y | UC | NA |
| Pearce, 2016 | Y | Y | N | N | Y | Y | Y | Y | N | NA |
| Wójcik, 2018 | N | Y | UC | N | UC | Y | UC | NA | NA | Y |
| Gokhale, 2018 | UC | Y | UC | N | Y | UC | UC | UC | UC | NA |
| Nilsen, 2018 | Y | Y | Y | N | Y | UC | Y | UC | UC | NA |
| Rodriguez-Muguruza, 2016 | N | Y | UC | N | Y | UC | Y | NA | UC | NA |
| Pamuk, 2013 | Y | Y | UC | UC | Y | UC | UC | UC | UC | NA |
| Jaffe, 2014 | Y | Y | Y | N | Y | UC | UC | UC | UC | NA |
| Jaffe, 2012 | Y | Y | Y | N | Y | UC | UC | UC | UC | NA |
| Watts, 2009 | N | Y | N | N | Y | Y | UC | Y | UC | NA |

Y, yes; N, no; N/A, not applicable; UC, unclear.

## List of publications included in the systematic literature review

1. Wójcik K, Kur-Zalewska J, Masiak A, et al. (2018) Polvas-retrospective registry of polish patients with anca-associated vasculitides. Ann Rheum Dis 77:1121-1122. <https://doi.org/10.1136/annrheumdis-2018-eular.1828>

2. Bell C, Shen Q, Sloane J, Katz A (2018) Clinical and Economic Characteristics of Patients Diagnosed with Eosinophilic Granulomatosis with Polyangiitis (EGPA, formerly Churg-Strauss Syndrome) in the United States. American Thoracic Society. In C41: HEALTH SERVICES RESEARCH IN PULMONARY DISEASE:A4951-A4951.

3. Gokhale M, Bell C, Doyle S, Fairburn-Beech J, Steinfeld J, Van Dyke MK (2018) Eosinophilc granulomatosis with polyangiitis (EGPA) prevalence and oral corticosteroid (OCS) use among asthma patients in a us commercial claims database. Am J Respir Crit Care Med. Epud ahead of print.

4. Nilsen AT, Watts RA, Koldingsnes W (2017) Epidemiology of ANCA-associated vasculitis in northern Norway. Ann Rheum Dis 76:319. <https://doi.org/10.1136/annrheumdis-2017-eular.4158>

5. Herlyn K, Gross WL, Reinhold-Keller E (2017) The incidence rates of ANCA-associated vasculitides in Northern Germany (schleswig-holstein) remain stable between 1998 to 2014. Ann Rheum Dis 76:325. <https://doi.org/10.1136/annrheumdis-2017-eular.3283>

6. Rodriguez-Muguruza S, Sanint J, Saenz-Sarda X, et al. (2016) Use of muscle biopsies for the diagnosis of systemic vasculitis in a rheumatology service. Arthritis and Rheumatology 68:3926-3928. <https://doi.org/10.1002/art.39977>

7. Pamuk Ö, Dönmez S, Calayir GB (2013) The incidences of anti-neutrophil cytoplasmic antibody-associated vasculitis in northeastern part of Turkey. Ann Rheum Dis 72. <https://doi.org/10.1136/annrheumdis-2013-eular.1892>

8. Fujimoto S, Watts RA, Kobayashi S, Suzuki K, Jayne DR, Scott DG, Hashimoto H, Nunoi H (2011) Comparison of the epidemiology of anti-neutrophil cytoplasmic antibody-associated vasculitis between Japan and the U.K. Rheumatology (Oxford) 50:1916-1920. <https://doi.org/10.1093/rheumatology/ker205>

9. Berti A, Cornec D, Crowson CS, Specks U, Matteson EL (2017) The Epidemiology of Antineutrophil Cytoplasmic Autoantibody-Associated Vasculitis in Olmsted County, Minnesota: A Twenty-Year US Population-Based Study. Arthritis & rheumatology (Hoboken, NJ) 69:2338-2350. <https://doi.org/10.1002/art.40313>

10. Kanecki K, Nitsch-Osuch A, Gorynski P, Tarka P, Tyszko P. 2017. p. 19-25.

11. Pamuk Ö N, Dönmez S, Calayır GB, Pamuk GE (2016) The epidemiology of antineutrophil cytoplasmic antibody-associated vasculitis in northwestern Turkey. Clin Rheumatol 35:2063-2071. <https://doi.org/10.1007/s10067-016-3232-y>

12. Nesher G, Ben-Chetrit E, Mazal B, Breuer GS (2016) The incidence of primary systemic vasculitis in Jerusalem: A 20-year hospital-based retrospective study. J Rheumatol 43:1072-1077. <https://doi.org/10.3899/jrheum.150557>

13. Romero-Gómez C, Aguilar-García JA, García-de-Lucas MD, Cotos-Canca R, Olalla-Sierra J, García-Alegría JJ, Hernández-Rodríguez J (2015) Epidemiological study of primary systemic vasculitides among adults in southern Spain and review of the main epidemiological studies. Clin Exp Rheumatol 33:S-11-18.

14. Jaffe A, Laverty A, Glaspole I, Pivot O (2014) Report of the australasian registry network for orphan lung disease (ARNOLD)-the first four years. Respirology 19:91. <https://doi.org/10.1111/resp.12263>

15. Sada KE, Amano K, Uehara R, Yamamura M, Arimura Y, Nakamura Y, Makino H (2014) A nationwide survey on the epidemiology and clinical features of eosinophilic granulomatosis with polyangiitis (Churg-Strauss) in Japan. Mod Rheumatol 24:640-644. <https://doi.org/10.3109/14397595.2013.857582>

16. Herlyn K, Buckert F, Gross WL, Reinhold-keller E (2014) Doubled prevalence rates of ANCA-associated vasculitides and giant cell arteritis between 1994 and 2006 in northern Germany. Rheumatology (United Kingdom) 53:882-889. <https://doi.org/10.1093/rheumatology/ket440>

17. Jaffe A, Laverty A, Glaspole I (2012) Report of the australasian registry network for orphan lung disease (arnold)-The first 2 years. Respirology 17:73. <https://doi.org/10.1111/j.1440-1843.2012.02143.x>

18. Mohammad A, Segelmark M (2011) Prevalence of ANCA associated vasculitis and polyarteritis nodosa in southern sweden-revisited 2010. Arthritis Rheum 63.

19. Vinit J, Muller G, Bielefeld P, Pfitzenmeyer P, Bonniaud P, Lorcerie B, Besancenot JF (2011) Churg-Strauss syndrome: Retrospective study in Burgundian population in France in past 10 years. Rheumatol Int 31:587-593. <https://doi.org/10.1007/s00296-009-1275-y>

20. Mohammad AJ, Jacobsson LTH, Westman KWA, Sturfelt G, Segelmark M (2009) Incidence and survival rates in Wegener's granulomatosis, microscopic polyangiitis, Churg-Strauss syndrome and polyarteritis nodosa. Rheumatology 48:1560-1565. <https://doi.org/10.1093/rheumatology/kep304>

21. Watts R, Mooney J, Scott D, Macgregor A (2009) A 20-year study of the epidemiology of ANCA-associated vascuitis (AAV) in the united kingdom. APMIS 117:159. <https://doi.org/10.1111/j.1600-0463.2009.02495.x>

22. Ormerod AS, Cook MC (2008) Epidemiology of primary systemic vasculitis in the Australian Capital Territory and south-eastern New South Wales. Intern Med J 38:816-823. <https://doi.org/10.1111/j.1445-5994.2008.01672.x>

23. Herlyn K, Hellmich B, Gross WL, Reinhold-Keller E (2008) Stable incidence of systemic vasculitides in Schleswig-Holstein, Germany. Deutsches Arzteblatt 105:355-361. <https://doi.org/10.3238/arztebl.2008.0355>

24. Mohammad AJ, Jacobsson LTH, Mahr AD, Sturfelt G, Segelmark M (2007) Prevalence of Wegener's granulomatosis, microscopic polyangiitis, polyarteritis nodosa and Churg-Strauss syndrome within a defined population in southern Sweden. Rheumatology 46:1329-1337. <https://doi.org/10.1093/rheumatology/kem107>

25. Mahr A, Guillevin L, Poissonnet M, Aymé S (2004) Prevalences of Polyarteritis Nodosa, Microscopic Polyangiitis, Wegener's Granulomatosis, and Churg-Strauss Syndrome in a French Urban Multiethnic Population in 2000: A Capture-Recapture Estimate. Arthritis Care Res 51:92-99.

26. Gonzalez-Gay MA, Garcia-Porrua C, Guerrero J, Rodriguez-Ledo P, Llorca J (2003) The epidemiology of the primary systemic vasculitides in northwest Spain: Implications of the Chapel Hill Consensus Conference definitions. Arthritis Care Res 49:388-393.

27. Watts RA, Gonzalez-Gay MA, Lane SE, Garcia-Porrua C, Bentham G, Scott DGI (2001) Geoepidemiology of systemic vasculitis: Comparison of the incidence in two regions of Europe. Ann Rheum Dis 60:170-172. <https://doi.org/10.1136/ard.60.2.170>

28. Watts RA, Lane SE, Bentham G, Scott DGI (2000) Epidemiology of systemic vasculitis: A ten-year study in the United Kingdom. Arthritis Rheum 43:414-419. [https://doi.org/10.1002/1529-0131(200002)43:2<414::AID-ANR23>3.0.CO;2-0](https://doi.org/10.1002/1529-0131(200002)43:2%3c414::AID-ANR23%3e3.0.CO;2-0)

29. Martin RM, Wilton LV, Mann RD (1999) Prevalence of Churg-Strauss syndrome, vasculitis, eosinophilia and associated conditions: Retrospective analysis of 58 prescription-event monitoring cohort studies. Pharmacoepidemiol Drug Saf 8:179-189. [https://doi.org/10.1002/(SICI)1099-1557(199905/06)8:3<179::AID-PDS414>3.0.CO;2-K](https://doi.org/10.1002/(SICI)1099-1557(199905/06)8:3%3c179::AID-PDS414%3e3.0.CO;2-K)

30. Haugeberg G, Bie R, Bendvold A, Storm Larsen A, Johnsen V (1998) Primary vasculitis in a Norwegian community hospital: A retrospective study. Clin Rheumatol 17:364-368. <https://doi.org/10.1007/BF01450893>

31. Watts RA, Carruthers DM, Scott DGI (1995) Epidemiology of systemic vasculitis: Changing incidence or definition? Semin Arthritis Rheum 25:28-34. <https://doi.org/10.1016/S0049-0172(95)80015-8>

32. Pearce FA, Lanyon PC, Grainge MJ, Shaunak R, Mahr A, Hubbard RB, Watts RA (2016) Incidence of ANCA-associated vasculitis in a UK mixed ethnicity population. Rheumatology (United Kingdom) 55:1656-1663. <https://doi.org/10.1093/rheumatology/kew232>

33. Reinhold-Keller E, Herlyn K, Wagner-Bastmeyer R, Gutfleisch J, Peter HH, Raspe HH, Gross WL (2002) No difference in the incidences of vasculitides between north and south Germany: First results of the German vasculitis register. Rheumatology 41:540-549.

34. Dadoniene J, Kirdaite G, Mackiewicz Z, Rimkevicius A, Haugeberg G (2005) Incidence of primary systemic vasculitides in Vilnius: a university hospital population based study. Ann Rheum Dis 64:335-336. <https://doi.org/10.1136/ard.2004.022335>

35. Reinhold-Keller E, Zeidler A, Gutfleisch J, Peter HH, Raspe HH, Gross WL (2000) Giant cell arteritis is more prevalent in urban than in rural populations: results of an epidemiological study of primary systemic vasculitides in Germany. Rheumatology (Oxford) 39:1396-1402. <https://doi.org/10.1093/rheumatology/39.12.1396>

36. Whyte AF, Sinkar S, Kette F, Smith W, Hissaria P (2011) Characteristics of churg-strauss syndrome in South Australia. Intern Med J 41:22.

37. Haq SA, Islam N, Abdal SJ, Ahmed AM, Islam MA, Ali SM, Azad AK (2013) The experience of treatment of vasculitis in Bangladesh. Int J Rheum Dis 16:22.

38. Kawano-Dourado L, De Oliveira Filho JB, Lima RM, Tavares MS, Barbas CSV (2017) Rituximab for refractory granulomatosis with polyangiitis and for eosinophilic granulomatosis with polyangiitis. Am J Respir Crit Care Med 195. <https://doi.org/10.1164/ajrccm-conference.2017.D23>

39. Pulenzas N, Carette S, Pagnoux C (2018) Conventional immunosuppressants for the treatment of patients with refractory or relapsing eosinophilic granulomatosis with polyangiitis. J Rheumatol 45:1016. <https://doi.org/10.3899/jrheum.180300>

40. Pulenzas N, Carette S, Pagnoux C (2018) Description of a Canadian center's cohort of 110 patients with eosinophilic granulomatosis with polyangiitis. J Rheumatol 45:1015-1016. <https://doi.org/10.3899/jrheum.180300>

41. Cottin V, Bel E, Bottero P, et al. (2016) Respiratory manifestations of eosinophilic granulomatosis with polyangiitis (Churg-Strauss). Eur Respir J 48:1429-1441. <https://doi.org/10.1183/13993003.00097-2016>

42. Comarmond C, Pagnoux C, Khellaf M, et al. (2013) Eosinophilic granulomatosis with polyangiitis (Churg-Strauss): Clinical characteristics and long-term followup of the 383 patients enrolled in the French Vasculitis Study Group cohort. Arthritis Rheum 65:270-281. <https://doi.org/10.1002/art.37721>

43. Pagnoux C, Comarmond C, Khellaf M, Cordier JF, Hamidou M, Viallard JF, Maurier F (2011) Churgstrauss syndrome: Description and long-term follow-up of the 383 patients enrolled in the FVSG cohort. Arthritis Rheum 63.

44. Guilpain P, Pagnoux C, Cohen P, Mahr A, Puechal X, Cordier JF, Guillevin L (2009) Anti-myeloperoxidase antibodies-associated vasculitides: Characteristics and outcome - The experience of the French vasculitis study group. APMIS 117:135. <https://doi.org/10.1111/j.1600-0463.2009.02491.x>

45. Terrier B, Saadoun D, Sène D, Ghillani P, Amoura Z, Deray G, Fautrel B, Piette JC, Cacoub P (2009) Antimyeloperoxidase antibodies are a useful marker of disease activity in antineutrophil cytoplasmic antibody-associated vasculitides. Ann Rheum Dis 68:1564-1571. <https://doi.org/10.1136/ard.2008.094714>

46. Guillevin L, Cohen P, Gayraud M, Lhote F, Jarrousse B, Casassus P (1999) Churg-Strauss syndrome: Clinical study and long-term follow-up of 96 patients. Medicine 78:26-37. <https://doi.org/10.1097/00005792-199901000-00003>

47. Hot A, Guerry MJ, Smith R, Sivasothy P, Guillevin L, Merkel P, Jayne D (2013) A multicenter survey of rituximab for eosinophilic granulomatosis with polyangiitis (Churg-Strauss). Presse Med 42:698. <https://doi.org/10.1016/j.lpm.2013.02.109>

48. Seeliger B, Forster M, Happe J, Forberg T, Moeser A, Neumann T, Kroegel C (2017) Interferon-alpha for Induction and Maintenance of Remission in Eosinophilic Granulomatosis with Polyangiitis: A Single-center Retrospective Observational Cohort Study. J Rheumatol 44:806-814. <https://doi.org/10.3899/jrheum.160907>

49. Mahrhold J, Hellmich B, Csernok E (2018) Eosinophilic granulomatosis with polyangiitis: A monocentric cohort analysis of manifestations and relapses of anca-positive and ancanegative patients. Arthritis and Rheumatology 70:1956-1957. <https://doi.org/10.1002/art.40700>

50. Yathish GC, Joshi PN, Parikh T, et al. (2014) Churg-strauss syndrome: Experience over ten years. Indian Journal of Rheumatology 9:S11. <https://doi.org/10.1016/j.injr.2014.10.005>

51. Latorre M, Novelli F, Baldini C, Dente F, Seccia V, Bacci E, Di Franco A, Paggiaro P (2013) Systemic and airway activity indices in a group of 32 patients with eosinophilic granulomatosis with polyangitis (EGPA). American Thoracic Society. In B35: AIRWAY INFLAMMATION IN ASTHMA: FOCUS ON EOSINOPHILS, MAST CELLS AND BASOPHILS:A2673-A2673.

52. Bacciu A, Buzio C, Giordano D, Pasanisi E, Vincenti V, Mercante G, Grasselli C, Bacciu S (2008) Nasal polyposis in Churg-Strauss syndrome. Laryngoscope 118:325-329. <https://doi.org/10.1097/MLG.0b013e318159889d>

53. Bacciu A, Bacciu S, Mercante G, et al. (2006) Ear, nose and throat manifestations of Churg-Strauss syndrome. Acta Otolaryngol 126:503-509. <https://doi.org/10.1080/00016480500437435>

54. Seccia V, Baldini C, Latorre M, et al. (2018) Focus on the involvement of the nose and paranasal sinuses in Eosinophilic Granulomatosis with polyangiitis (Churg-Strauss Syndrome): Nasal cytology reveals infiltration of eosinophils as a very common feature. Int Arch Allergy Immunol 175:61-69. <https://doi.org/10.1159/000484602>

55. Bottero P, Bonini M, Vecchio F, Grittini A, Patruno GM, Colombo B, Sinico RA (2007) The common allergens in the Churg-Strauss syndrome. Allergy: European Journal of Allergy and Clinical Immunology 62:1288-1294. <https://doi.org/10.1111/j.1398-9995.2007.01486.x>

56. Pavone L, Grasselli C, Chierici E, et al. (2006) Outcome and prognostic factors during the course of primary small-vessel vasculitides. J Rheumatol 33:1299-1306.

57. Latorre M, Seccia V, Baldini C, et al. (2015) Nasal disease in chronic severe asthma (CSA) and eosinophilic granulomatosis with poliangiitis (EGPA). Eur Respir J 46: PA3588. <https://doi.org/10.1183/13993003.congress2015.PA3588>

58. Latorre M, Baldini C, Novelli F, Dente F, Bombardieri S, Paggiaro PL (2012) Airway and systemic inflammation during Churg-Strauss syndrome natural course. Eur Respir J 40: 1836.

59. Latorre M, Baldini C, Novelli F, Dente F, Grosso S, Della Rossa P, Cianchetti S, Bombardieri S, Paggiaro P (2012) Airway inflammation and systemic inflammation in Churg-Strauss syndrome: Two faces of disease. American Thoracic Society. In C33: RHINITIS, SINUSITIS AND OTHER COMORBIDITIES:A4207.

60. Latorre M, Baldini C, Novelli F, Dente F, Seccia V, Paggiaro PL (2013) Allergic eosinophilic granulomatosis with polyangiitis: Evidence for disease subtypes? Allergy: European Journal of Allergy and Clinical Immunology 68:76-77. <https://doi.org/10.1111/all.12249>

61. Baldini C, Pepe P, Latorre M, et al. (2013) THU0205 The temporal appearance of symptoms and signs in churg strauss syndrome: From the prodromal phase to damage. Annals of the Rheumatic Disease 71:244. <https://doi.org/10.1136/annrheumdis-2012-eular.2170>

62. Baldini C, Grossi S, Latorre M, Pepe P, Giorgerini V, Rossa AD, Dente F (2011) Airway inflammation and systemic inflammation during churg-strauss syndrome natural course: A multidisciplinary monocentric crosse-sectional study. Arthritis Rheum 63.

63. Latorre M, Baldini C, Seccia V, Novelli F, Bombardieri S, Paggiaro PL (2013) Clinical assessment of asthma severity partially corresponds to sputum eosinophilic airway inflammation in allergic eosinophilic granulomatosis with polyangiitis. Presse Med 42:697. <https://doi.org/10.1016/j.lpm.2013.02.107>

64. Baldini C, Latorre M, Seccia V, Della Rossa A, Tavoni A, Paggiaro PL, Sellari Franceschini S, Bombardieri S (2013) Eosinophilic granulomatosis with polyangitis (EGPA): Clinical and immunologic expression in a single center cohort. Presse Med 42:701. <https://doi.org/10.1016/j.lpm.2013.02.115>

65. Jeannin G, Salviani C, Possenti S, Regazzoli A, Allegri F, Cancarini G, Gregorini GA (2013) Re-treatment with rituximab in ANCA-associated vasculitis only in the presence of clinical relapse: A single centre experience. Presse Med 42:776. <https://doi.org/10.1016/j.lpm.2013.02.295>

66. Baldini C, Seccia V, Latorre M, et al. (2012) Rhinosinusits and nasal polyps in the diagnosis and follow up of patients with eosinophilic granulomatosis with polyangitis (ex-churg strauss syndrome). Arthritis Rheum 64:S661-S662. <https://doi.org/10.1002/art.37735>

67. Watanabe H, Sada KE, Matsumoto Y, et al. (2018) Association Between Reappearance of Myeloperoxidase–Antineutrophil Cytoplasmic Antibody and Relapse in Antineutrophil Cytoplasmic Antibody–Associated Vasculitis: Subgroup Analysis of Nationwide Prospective Cohort Studies. Arthritis and Rheumatology 70:1626-1633. <https://doi.org/10.1002/art.40538>

68. Saku A, Furuta S, Hiraguri M, et al. (2018) Longterm outcomes of 188 Japanese patients with eosinophilic granulomatosis with polyangiitis. J Rheumatol 45:1159-1166. <https://doi.org/10.3899/jrheum.171352>

69. Nakamoto K, Saraya T, Ogawa Y, Ishii H, Takizawa H (2018) Comparison of findings on thoracic computed tomography with the severity and duration of bronchial asthma in patients with eosinophilic granulomatosis with polyangiitis. Respir Med 139:101-105. <https://doi.org/10.1016/j.rmed.2018.05.003>

70. Tsurikisawa N, Oshikata C, Kinoshita A, Tsuburai T, Hiroshi Saito A (2017) Longterm prognosis of 121 patients with eosinophilic granulomatosis with polyangiitis in Japan. J Rheumatol 44:1206-1215. <https://doi.org/10.3899/jrheum.161436>

71. Saku A, Furuta S, Yamagata M, et al. (2017) Risk factors for vasculitis relapse in patients with eosinophilic granulomatosis with polyangiitis. Rheumatology (United Kingdom) 56:iii132. <https://doi.org/10.1093/rheumatology/kex133>

72. Hasegawa W, Yamauchi Y, Yasunaga H, Sunohara M, Jo T, Matsui H, Fushimi K, Takami K, Nagase T (2015) Factors that predict in-hospital mortality in eosinophilic granulomatosis with polyangiitis. Allergy: European Journal of Allergy and Clinical Immunology 70:585-590. <https://doi.org/10.1111/all.12597>

73. Yoo J, Kim HJ, Ahn SS, Jung SM, Song JJ, Park YB, Lee SW (2017) Clinical and prognostic features of Korean patients with MPO-ANCA, PR3-ANCA and ANCA-negative vasculitis. Clin Exp Rheumatol 35:111-118.

74. Lee C, Lee B, Lee J, Choi D (2012) Churg-strauss syndrome: The clinical features and long-term prognosis of 47 patients. J Allergy Clin Immunol 129:AB57. <https://doi.org/10.1016/j.jaci.2011.12.708>

75. Sokołowska B, Szczeklik W, Włudarczyk A, Kuczia P, Jakieła B, Gasior J, Bartyzel S, Rewerski P, Musiał J (2014) ANCA-positive and ANCA-negative phenotypes of eosinophilic granulomatosis with polyangiitis (EGPA): Outcome and long-term follow-up of 50 patients from a single polish centre. Clin Exp Rheumatol 32:S41-S47.

76. Sokołowska B, Szczeklik W, Mastalerz L, Kuczia P, Wodkowski M, Stodółkiewicz E, MacIoł K, Musiał J (2013) Effect of delayed diagnosis on disease course and management of Churg-Strauss syndrome: A retrospective study. Clin Rheumatol 32:349-354. <https://doi.org/10.1007/s10067-012-2127-9>

77. Szczeklik W, Sokołowska BM, Zuk J, Mastalerz L, Szczeklik A, Musiał J (2011) The course of asthma in Churg-Strauss syndrome. J Asthma 48:183-187. <https://doi.org/10.3109/02770903.2010.551796>

78. Wójcik K, Wawrzycka-Adamczyk K, Włudarczyk A, et al. (2019) Clinical characteristics of Polish patients with ANCA-associated vasculitides—retrospective analysis of POLVAS registry. Clin Rheumatol. <https://doi.org/10.1007/s10067-019-04538-w>

79. Kim MY, Sohn KH, Song WJ, Park HW, Cho SH, Min KU, Kang HR (2014) Clinical features and prognostic factors of Churg-Strauss syndrome. Korean J Intern Med 29:85-95. <https://doi.org/10.3904/kjim.2014.29.1.85>

80. Solans-Laqué R, Fraile G, Castillo MJ, et al. (2014) Eosinophilic granulomatosis with poliangeitis (EGPA): Clinical features and outcome in a large serie of spanish patients. Ann Rheum Dis 73. <https://doi.org/10.1136/annrheumdis-2014-eular.4714>

81. Solans-Laqué R, Fraile G, Rodriguez-Carballeira M, et al. (2017) Clinical characteristics and outcome of Spanish patients with ANCA-associated vasculitides Impact of the vasculitis type, ANCA specificity, and treatment on mortality and morbidity. Medicine (United States) 96. <https://doi.org/10.1097/MD.0000000000006083>

82. Armengot M, Garcia-Lliberos A, Gomez MJ, Navarro A, Martorell A (2013) Sinonasal involvement in systemic vasculitides and cocaine-induced midline destructive lesions: Diagnostic controversies. Allergy & rhinology (Providence, RI) 4:e94-99. <https://doi.org/10.2500/ar.2013.4.0051>

83. Solans R, Bosch JA, Ṕerez-Bocanegra C, Selva A, Huguet P, Alijotas J, Orriols R, Armandans L, Vilardell M (2001) Churg-Strauss syndrome: Outcome and long-term follow-up of 32 patients. Rheumatology 40:763-771.

84. Yilmaz I, Çelik G, Aydin O, et al. (2015) Churg-Strauss syndrome: A new endotype of severe asthma? Results of 14 Turkish patients. Clin Respir J 9:350-358. <https://doi.org/10.1111/crj.12154>

85. Martin-Suarez I, D'Cruz D, Mansoor M, Fernandes AP, Khamashta MA, Hughes GR (1997) Immunosuppressive treatment in severe connective tissue diseases: effects of low dose intravenous cyclophosphamide. Ann Rheum Dis 56:481-487.

86. Reid AJC, Harrison BDW, Watts RA, Watkin SW, McCann BG, Scott DGI (1998) Churg-Strauss syndrome in a district hospital. QJM - Monthly Journal of the Association of Physicians 91:219-229.

87. Durel CA, Berthiller J, Caboni S, Jayne D, Ninet J, Hot A (2016) Long-Term Followup of a Multicenter Cohort of 101 Patients with Eosinophilic Granulomatosis with Polyangiitis (Churg-Strauss). Arthritis Care Res 68:374-387. <https://doi.org/10.1002/acr.22686>

88. Rhee RL, Dehghan N, Sreih AG, et al. (2017) Late-onset relapse in patients with systemic vasculitis. Rheumatology (United Kingdom) 56:iii107-iii108. <https://doi.org/10.1093/rheumatology/kex128>

89. Rhee RL, Dehghan N, Sreih AG, et al. (2016) Late-onset relapse in patients with systemic vasculitis. Arthritis and Rheumatology 68:3954-3956. <https://doi.org/10.1002/art.39977>

90. Keogh KA, Specks U (2003) Churg-Strauss syndrome: clinical presentation, antineutrophil cytoplasmic antibodies, and leukotriene receptor antagonists. Am J Med 115:284-290.

91. Berti A, Volcheck GW, Cornec D, Smyth RJ, Specks U, Keogh KA (2018) Severe/uncontrolled asthma and overall survival in atopic patients with eosinophilic granulomatosis with polyangiitis. Respir Med 142:66-72. <https://doi.org/10.1016/j.rmed.2018.07.017>

92. Santos-Pinheiro F, Li Y (2015) Eosinophilic granulomatosis with polyangiitis presenting with polyneuropathy-a case series. Neurology 84.

93. Abu-Shakra M, Smythe H, Lewtas J, Badley E, Weber D, Keystone E (1994) Outcome of polyarteritis nodosa and Churg-Strauss syndrome: An analysis of twenty-five patients. Arthritis Rheum 37:1798-1803. <https://doi.org/10.1002/art.1780371214>

94. Bell CF, Blauer-Peterson C, Mao J (2018) Burden of illness associated with eosinophilic granulomatosis with polyangiitis (EGPA, formerly churg-strauss syndrome): Evidence from a managed care database in the United States. Arthritis and Rheumatology 70:1951-1952. <https://doi.org/10.1002/art.40700>

95. Denis L, Samson M, Maurier F, et al. (2017) Off-label use of biological therapies in relapsing and/or refractory eosinophilic granulomatosis with polyangiitis (churg-strauss). Arthritis and Rheumatology 69.

96. Zagvozdkina E, Novikov P, Moiseev S (2017) Clinical presentation and outcomes of eosinophilic granulomatosis with polyangiitis: ANCA-negative versus ANCA-positive. Ann Rheum Dis 76:331. <https://doi.org/10.1136/annrheumdis-2017-eular.5259>

97. Seeliger B, Foerster M, Moeser A, Happe J, Kroegel C, Neumann T (2014) Efficacy and safety of IFN-alpha in induction and maintenance of remission in patients with eosinophilic granulomatosis with polyangiitis (EGPA). Single center observational study. Arthritis and Rheumatology 66:S787. <https://doi.org/10.1002/art.38914>

98. Zagvozdkina E, Moiseev S, Novikov P (2017) Clinical and laboratory feature of relapses in patients with eosinophilic granulomatosis with polyangiitis. Rheumatology (United Kingdom) 56:iii132. <https://doi.org/10.1093/rheumatology/kex133>

99. Zagvozdkina E, Novikov P, Moiseev S (2017) ANCA-negative and ANCA-positive eosinophilic granulomatosis with polyangitis: Differences in clinical presentation but similar outcomes. Rheumatology (United Kingdom) 56:iii131-iii132. <https://doi.org/10.1093/rheumatology/kex133>

100. Grayson PC, Monach PA, Pagnoux C, et al. (2015) Value of commonly measured laboratory tests as biomarkers of disease activity and predictors of relapse in eosinophilic granulomatosis with polyangiitis. Rheumatology (United Kingdom) 54:1351-1359. <https://doi.org/10.1093/rheumatology/keu427>
